# Extended N-Terminal Acetyltransferase Naa50 in Filamentous Fungi Adds to Naa50 Diversity

**DOI:** 10.3390/ijms231810805

**Published:** 2022-09-16

**Authors:** Jonas Weidenhausen, Jürgen Kopp, Carmen Ruger-Herreros, Frank Stein, Per Haberkant, Karine Lapouge, Irmgard Sinning

**Affiliations:** 1 Heidelberg University Biochemistry Center (BZH), 69120 Heidelberg, Germany; 2 Center for Molecular Biology of the University of Heidelberg (ZMBH), 69120 Heidelberg, Germany; 3 Proteomics Core Facility, EMBL Heidelberg, 69117 Heidelberg, Germany; 4 Protein Expression and Purification Core Facility, EMBL Heidelberg, 69117 Heidelberg, Germany

**Keywords:** N-terminal acetyltransferase, NAT, Naa50, NatE, GNAT domain, *Chaetomium thermophilum*, *Neurospora crassa*, ribosome association, dynein light chain protein 1, X-ray structure

## Abstract

Most eukaryotic proteins are N-terminally acetylated by a set of Nα acetyltransferases (NATs). This ancient and ubiquitous modification plays a fundamental role in protein homeostasis, while mutations are linked to human diseases and phenotypic defects. In particular, Naa50 features species-specific differences, as it is inactive in yeast but active in higher eukaryotes. Together with NatA, it engages in NatE complex formation for cotranslational acetylation. Here, we report Naa50 homologs from the filamentous fungi *Chaetomium thermophilum* and *Neurospora crassa* with significant N- and C-terminal extensions to the conserved GNAT domain. Structural and biochemical analyses show that *Ct*Naa50 shares the GNAT structure and substrate specificity with other homologs. However, in contrast to previously analyzed Naa50 proteins, it does not form NatE. The elongated N-terminus increases Naa50 thermostability and binds to dynein light chain protein 1, while our data suggest that conserved positive patches in the C-terminus allow for ribosome binding independent of NatA. Our study provides new insights into the many facets of Naa50 and highlights the diversification of NATs during evolution.

## 1. Introduction

The amino termini of proteins are subject to a wide range of enzymatic processing, which is essential for their function, regulation, and protein homeostasis [1]. N-terminal acetylation is among the most important and ubiquitous co- and post-translational modifications in archaea, bacteria, and eukaryotes [2]. Up to 60% of yeast proteins and 80–90% of human and plant proteins are irreversibly N-terminally acetylated by a set of Nα acetyltransferases (NATs) [3,4,5]. These enzymes transfer an acetyl group from acetyl coenzyme A (AcCoA) onto the positively charged amino terminus of target proteins and thereby change the chemical properties of the N-terminus [6]. This modification affects various biological functions such as protein folding, protein stability, protein degradation, protein complex formation, and protein targeting [7,8,9,10,11,12]. Dysfunctions of NATs are linked to human diseases such as cancer, developmental diseases, and Ogden syndrome as well as immunity and drought-stress tolerance in plants [7,13,14,15,16,17].

Eight NATs (NatA–NatH) have been identified in eukaryotes, and they consist of at least one catalytic subunit and optional additional subunits [7]. They differ in substrate specificity and localization. While NatA–NatE act on the ribosome to cotranslationally acetylate nascent chains, the membrane-bound NatF acetylates membrane proteins [18,19,20,21,22], and NatG acetylates proteins in chloroplasts [23]. NatH post-translationally acetylates acidic actin N-termini in animals with profilin as a potential chaperone for proper acetylation [24,25,26,27]. NatA–C and NatE are protein complexes that comprise at least one catalytic subunit (Naa10, Naa20, Naa30, and Naa10/Naa50, respectively) and additional auxiliary subunits [28,29,30,31,32,33,34]. NatD and NatF–H comprise only one catalytic Nα-acetyltransferase (Naa40 and Naa60–Naa80) [7]. The active subunits belong to the GCN5 (general control non-repressible 5)-related N-acetyltransferase (GNAT) superfamily with a conserved mixed α/β-fold, which features a relatively low sequence identity [35,36,37]. GNATs also contain one family of lysine Nε-acetyltransferases (KATs) [38]. NATs and KATs differ, especially in their respective substrate binding sites. NATs, such as *Arabidopsis thaliana* Naa50, have an extended β6–β7 loop that, together with the α1–α2 loop, folds over the N-terminal peptide substrates [39,40]. The substrate binding site of KATs is more open and allows for the binding of internal lysine substrates, as it has been structurally analyzed for the enzyme GCN5 with the histone H3 tail [41].

In humans, NatA acetylates 38%, NatB acetylates 21%, and NatC/E/F together acetylate 21% of all proteins sharing similar substrate specificities [2]. The NatE complex consists of Naa50 bound to the NatA complex (Naa10/Naa15). While Naa10 in the NatA complex has a substrate specificity for small residues (Ala, Cys, Gly, Ser, Thr, or Val) after initiator methionine (iMet) removal [3,32], Naa50 acetylates methionine when the second residue is hydrophobic/amphipathic (Ala, Ile, Leu, Lys, Met, Phe, Ser, Thr, Tyr, or Val) [42,43]. In yeast, Naa50 is stably bound to NatA, while in humans only 20% of Naa50 forms a complex with NatA [34,44]. Human and fission yeast Naa50 bind NatA with an affinity in the nanomolar range [45]. A knockout of Naa50 in human cells or mutations in Naa50 alleles in Drosophila both lead to improper sister chromatid separation [44,46,47]. Depletion studies of Naa50 in Arabidopsis showed an important role for development, growth, and stress responses [48,49,50]. In contrast, Naa50 deletion in *Saccharomyces cerevisiae* showed no specific phenotype [34]. Moreover, the N-terminal acetylome analysis of the yeast Naa50 deletion revealed reduced acetylation of only six NatA-type substrate proteins [43].

Recent structural and functional studies with Naa50 from different organisms suggest that conserved tyrosine and histidine residues are important for acetyltransferase activity through an ordered water molecule [39,51]. These two residues are absent in *S. cerevisiae* and *Schizosaccharomyces pombe* Naa50, which explains their inactivity and lack of deletion phenotype, although they retain the capability to bind (Ac)CoA [6,39,45]. Despite being inactive, *Sp*Naa50 mildly promotes *Sp*NatA efficiency (k_cat_/K_m_) in the NatE complex [45]. Human Naa50 decreases the k_cat_ of Naa10 in the NatE complex, and Naa50 and Naa10 promote each other’s acetylation efficiencies [45,52]. Structural studies of the human and *S. cerevisiae* NatE complexes revealed that Naa50 mostly binds to Naa15 and has only a minor interaction with Naa10 (PDB identifier: 4XNH; unpublished data) [45,52]. In general, the Naa50–Naa15 interface is smaller than the Naa10–Naa15 interface, where Naa15 engulfs Naa10. *Sc*NatE binds to the ribosome close to the tunnel exit, with major interaction sites contributed by Naa15 (with ribosomal RNA expansion segments ES27 and ES39, RNA helices H24 and H47, and the ribosomal protein el31) as well as Naa50 (with ES7a), highlighting a role of inactive yeast Naa50 in ribosome binding [53]. 

In this study, we identify and characterize Naa50 from the filamentous fungi *Chaetomium thermophilum* and *Neurospora crassa*, which contain significant N- and C-terminal extensions. Unlike human or yeast Naa50, *Ct*Naa50 does not bind to NatA and may interact with the ribosome through C-terminal-positive patches. Our study highlights species specific adaptations and adds to the diversity of Naa50 proteins.

## 2. Results

### 2.1. Chaetomium thermophilum and Neurospora crassa Naa50 Have Elongated Termini

**Figure 1:**
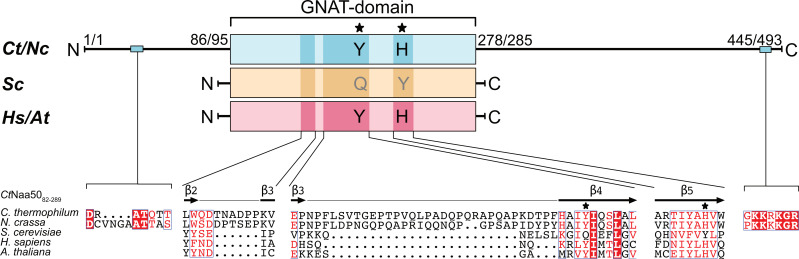
*Chaetomium thermophilum* and *Neurospora crassa* Naa50 have N- and C-terminal extensions. Naa50 from *C. thermophilum* (*Ct*), *N. crassa* (*Nc)*, *Saccharomyces cerevisiae* (*Sc*), *Homo sapiens* (*Hs*), and *Arabidopsis thaliana* (*At*) showed high conservation within the GNAT domain in a multiple sequence alignment. *Ct*Naa50 and *Nc*Naa50 (teal) have excessive N- and C-terminal extensions (*Ct*Naa50 residues 1-86 and 278-445, and *Nc*Naa50 residues 1-95 and 285-493, respectively) compared to yeast (yellow) and human/Arabidopsis (salmon) homologs. Secondary structure elements (β-strands) from a GNAT domain construct *Ct*Naa50_82-289_ are shown on top of the alignment. *Ct*Naa50 contains a dynein light chain 1 protein binding motif (RxTQT) in the N-terminal extension. Loops β2–β3 and β3–β4 are longer in *Ct* and *Nc*. *Ct*/*Nc*Naa50 feature a conserved positive patch KK(R/K)KgR at the C-terminus. Tyrosine and histidine residues (★) are catalytic residues and are absent in yeast. The sequence alignment was performed using Clustal Omega and visualized using ESPript 3.0 [56,57]. Fully conserved residues are represented as white letters in red boxes. Similarities are shown with red letters in blue frames.

Naa50 orthologs have been identified across the eukaryotic kingdom in a study that highlights that NatA–NatF were likely present in the last eukaryotic common ancestor (LECA) and did not diversify much during eukaryotic evolution [54]. The human Naa50 protein sequence (UniProt identifier Q9GZZ1) was used in a BLAST search to identify orthologous proteins in *Neurospora crassa* (*Nc*) and *Chaetomium thermophilum* (*Ct*). Two candidates were found: The *Neurospora crassa* “hypothetical protein NCU00576” (NCBI identifier XP_964737.1; E-score value = 2 × 10^−14^ with a sequence identity of 27%) and the *C. thermophilum* “N-acetyltransferase-like protein” (XP_006692007.1; E-score value = 3 × 10^−13^ with a sequence identity of 26%). Characterized Naa50 proteins from human, yeast, fruit fly, and Arabidopsis have a GNAT fold with only few amino acids added at the N- and C-termini [39,45,46,51]. Surprisingly, the two Naa50 candidates from *N. crassa* and *C. thermophilum* have more than twice the length compared to other organisms and consist of 494 and 445 amino acids (Figure 1, Supplementary Figure S1). In both proteins, the putative GNAT domain (residues 86 to 278 in *C. thermophilum* or 95 to 285 in *N crassa*) is surrounded by long N- and C-terminal extensions, which are predicted to be unstructured. Typically, extensions of the GNAT domain serve a function as, for example, the N-terminal extension of Naa40 (NatD), which wraps around the protein for stabilization [55], and the C-terminal helix of Naa60 (NatF), which is required for membrane association [20,21]. Using the *Ct*Naa50 candidate in a BLAST search highlighted numerous elongated putative Naa50 proteins in the fungal Sordariomycetes class. Multiple sequence alignment with selected Naa50 candidates of *N. crassa*, *C. thermophilum*, *Chaetomium globosum* (XP_001222293.1; “hypothetical protein CHGG_06198”), *Thermothelomyces thermophilus* (XP_003659866.1; “uncharacterized protein MYCTH_2297367”), *Neurospora tetrasperma* (GenBank ID: EGZ76581.1; “hypothetical protein NEUTE2DRAFT_98549”), *Daldinia childiae* (XP_033432349.1; “N-alpha-acetyltransferase 50”), and *Phaeoacremonium minimum* (XP_007919293.1; “putative gcn5-related n acetyltransferase protein”) revealed that the C- and N-terminal extensions have a high degree of conservation (Supplementary Figure S2). The *Ct*Naa50 candidate shares overall sequence identities of 44.4–58.2% with the other fungal proteins.

### 2.2. Deletion of naa50 or NatA Genes Leads to Neurospora crassa Growth Defects

**Figure 2:**
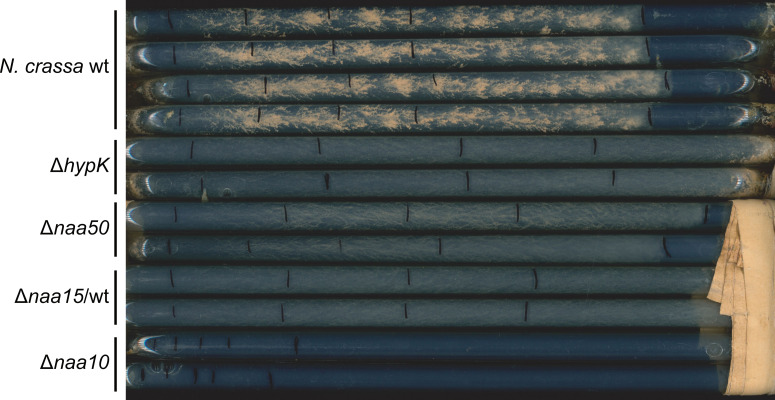
Knockout of *naa50* and NatA subunits resulted in a *Neurospora crassa* growth phenotype. The wild-type (wt) strain formed typical conidia under light conditions at room temperature. Knocking out *hypK*, *naa50*, *naa15* (heterokaryon), and *naa10* led to improper conidia formation and different growth patterns. Front growth was marked after every 24 h for 5 days.

*N. crassa* has been widely used over the past decades as a model organism to study genetics and the circadian rhythm [58]. We used race tubes to study the phenotype of different knockouts on *N. crassa* growth. In addition to the *naa50* (NCU00576) knockout, we characterized the phenotypes of knockouts of *Nc*NatA subunits (*naa10*: NCU08944; *naa15*: NCU07936; and *hypK*: NCU02784) (Figure 2). As the knockout of *naa15* is lethal, we used the heterokaryon strain (Δ*naa15*/wt) in this study. Compared to wild-type (wt) *N. crassa*, all knockout strains produced less or no conidia during growth at full light and room temperature in the race tubes. The *naa50* knockout (Δ*naa50*) led to a growth phenotype different from the wild type, as it grew slightly faster than the wild type over a period of five days without forming as many conidia. The *hypK* knockout (Δ*hypK*) or *naa15* heterokaryon (Δ*naa15*/wt) resulted in a similar phenotype with faster growth than the wild type and Δ*naa50* but without forming conidia. The *naa10* knockout (Δ*naa10*) had the most severe phenotype, as it did not form conidia and grew only slightly. Taken together, the *naa50* knockout had a less severe phenotype than the NatA subunits. Nevertheless, knockout of the identified *Nc*Naa50 protein resulted in a phenotypic effect, albeit milder compared to the NatA subunits, highlighting that this elongated Naa50 is important for normal growth in vivo.

### 2.3. Naa50 Acetylates Canonical NatC/E/F-Type Substrates

To characterize the NAT activity, *Ct*Naa50_82-289_ and *Nc*Naa50_93-287_, both corresponding to the predicted GNAT domains without the unstructured N- and C-termini, were expressed in *E. coli*. Both could be purified to homogeneity, and their substrate specificities and Michaelis–Menten kinetic parameters were analyzed using in vitro acetylation assays. Short canonical substrate peptides, previously tested with other NATs, were used to determine the substrate specificity: NatA substrate SESS [3], NatB substrate MDEL [59], and NatC/E/F substrates MASS, MLGTE, and MVNALE [39,60].

**Figure 3:**
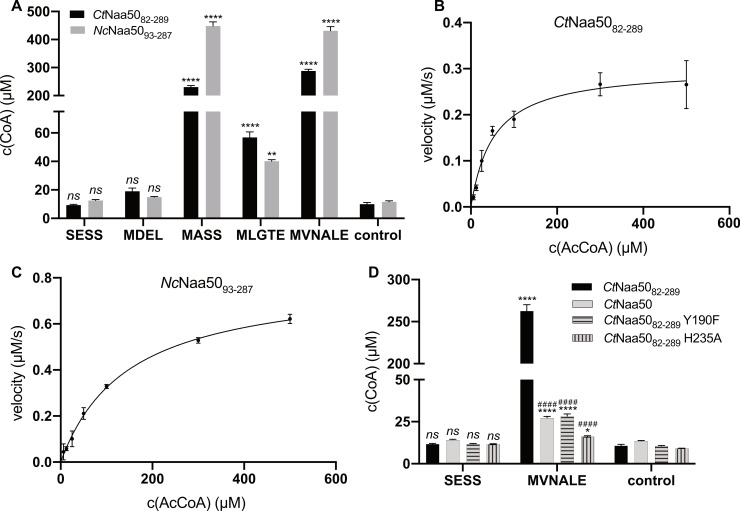
*Ct*Naa50_82-289_ and *Nc*Naa50_93-287_ acetylate canonical NatC/E/F substrates. (**A**) Both GNAT constructs, *Ct*Naa50_82-289_ and *Nc*Naa50_93-287_, showed the highest acetylation activity towards MVNALE and MASS peptides, followed by MLGTE and insignificant activity for MDEL. SESS was not acetylated. Control reactions were performed without peptides. Acetylation by both enzymes of different peptides was compared against the peptide-free controls with a one-way ANOVA statistical analysis with Dunnett’s correction (*ns* = not significant, ** = *p*-value ≤ 0.0021, **** = *p*-value ≤ 0.0001). (**B**) Michaelis–Menten kinetics of MVNALE acetylation by *Ct*Naa50_82-289_. (**C**) Michaelis–Menten kinetics of MVNALE acetylation by *Nc*Naa50_93-287_. (**D**) *Ct*Naa50_82-289_ Y190F and H235A mutants showed reduced MVNALE acetylation. Full-length *Ct*Naa50 also showed reduced activity. Acetylation of SESS or MVNALE by *Ct*Naa50 variants was compared against the peptide-free controls with a one-way ANOVA with Dunnett’s correction (*ns* = not significant, * = *p*-value ≤ 0.0332, **** = *p*-value ≤ 0.0001). Acetylation of MVNALE by *Ct*Naa50 variants was compared against *Ct*Naa50_82-289_ with a one-way ANOVA with Dunnett’s correction (*####* = *p*-value ≤ 0.0001). All measurements were performed in triplicates and are presented as means ± SD.

*Ct*Naa50_82-289_ showed its highest acetylation activity towards MVNALE, followed by the MASS peptide (Figure 3A). MLGTE was acetylated to a lesser extent, and MDEL was only slightly acetylated, while SESS was not acetylated. *Ct*Naa50_82-289_ and *Nc*Naa50_93-287_ acetylated identical substrates. *Nc*Naa50_93-287_ acetylated MVNALE and MASS the most, and MLGTE was only slightly acetylated (Figure 3A). MDEL and SESS were not acetylated. We used the best substrate, MVNALE, to determine the Michaelis–Menten kinetics of *Ct*Naa50_82-289_ and *Nc*Naa50_93-287_ for AcCoA (Figure 3B,C). *Ct*Naa50_82-289_ showed a higher affinity for AcCoA, with a Michaelis–Menten constant, K_m_, of 52.7 ± 8.6 μM, compared to *Nc*Naa50_93-287_, with a K_m_ of 146.9 ± 12.1 μM. However, *Ct*Naa50_82-289_ was less active, with a turnover number, k_cat_, of 36.4 ± 1.8 min^−1^, compared to *Nc*Naa50_93-287_, with a k_cat_ of 95.9 ± 3.0 min^−1^. Both have the same acetylation efficiency (0.69 ± 0.12 min^−1^µM^−1^ for *Ct*Naa50_82-289_ or 0.65 ± 0.06 min^−1^µM^−1^ for *Nc*Naa50_93-287,_ respectively) (Table 1). Compared to previously reported human Naa50 parameters [61], both have a slightly lower affinity for AcCoA but are more active (*Hs*Naa50: k_cat_ = 5.22 ± 0.23 min^−1^ and K_m_ = 4.62 ± 0.85 µM with a peptide starting with MLGPE). In comparison, *Arabidopsis thaliana* Naa50 was less active for its best substrate, MASS [39].

To study the effect of both elongated termini on acetylation, we performed in vitro acetylation of the MVNALE peptide with N-terminal (*Ct*Naa50_82-445_, expressed in *E. coli*) and C-terminal (*Ct*Naa50_1-289_, expressed in *S. cerevisiae*) truncation variants (Supplementary Figure S3A,B). Our data show that the terminal extensions had only a minor impact on the acetylation efficiency compared to the *Ct*Naa50_82-289_ GNAT domain. *Ct*Naa50_82-445_, containing 156 additional C-terminal amino acids, had a slightly reduced efficiency (84 ± 13% of *Ct*Naa50_82-289_) compared to the GNAT construct *Ct*Naa50_82-289_, while *Ct*Naa50_1-289_, containing 81 additional N-terminal amino acids had a reduced efficiency (74 ± 10%). Both extensions resulted in a slight decrease in acetylation efficiency compared to the GNAT construct.

The Naa50 active site generally contains histidine and tyrosine residues, which bind a catalytically important water molecule and are important for acetylation [39,51]. We created two *Ct*Naa50_82-289_ mutants (Y190F and H235A) to confirm that these residues are also required for acetylation in *C. thermophilum*. Both mutants showed reduced acetylation of MVNALE but were not completely inactive (Figure 3D, Supplementary Figure S3C,D). H235A had a higher impact on acetylation than Y190F (Table 1). Similar to *Ct*Naa50_82-289_, the affinity of both mutants for AcCoA was in the lower micromolar range (K_m_ = 22.0 ± 4.0 µM for Y190F and 15.3 ± 3.1 µM for H235A). However, the turnover numbers were decreased (k_cat_ = 5.6 ± 0.3 min^−1^ for Y190F and 1.4 ± 0.1 min^−1^ for H235A). 

Full-length *Ct*Naa50 could only be expressed in and purified from *C. thermophilum* and could acetylate MVNALE but not SESS (Figure 3D). However, MVNALE acetylation was decreased compared to the *Ct*Naa50_82-289_ GNAT domain construct. The observed total acetylation was similar to the *Ct*Naa50_82-289_ Y190F mutant. The N- and C-terminal extensions significantly lowered the acetylation activity. 

### 2.4. The N-Terminus of CtNaa50 Stabilizes the GNAT Domain

**Figure 4:**
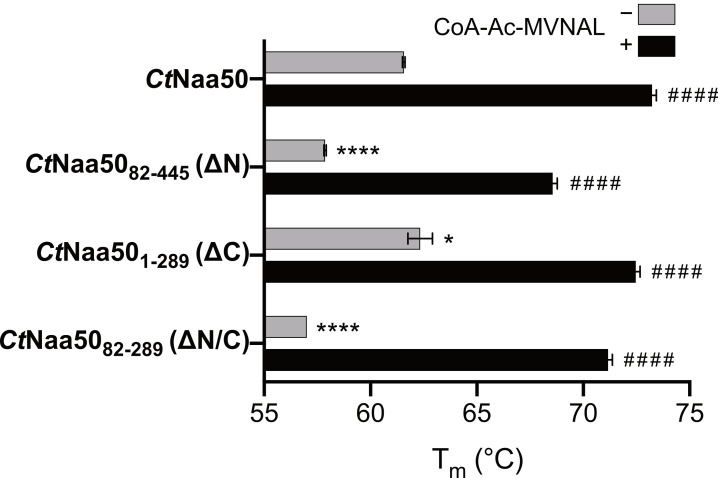
*Ct*Naa50 constructs containing the N-terminal extension have an increased thermostability. *Ct*Naa50 and *Ct*Naa50_1-289_ feature the highest melting temperatures without the bisubstrate analog CoA-Ac-MVNAL. All constructs bind CoA-Ac-MVNAL, as highlighted by a dramatic increase in thermostability when incubated with tenfold molar excess. The melting temperatures of *Ct*Naa50 variants without CoA-Ac-MVNAL were compared against *Ct*Naa50 with a one-way ANOVA statistical analysis with Dunnett’s correction (* = *p*-value ≤ 0.0332, **** = *p*-value ≤ 0.0001). The melting temperatures with CoA-Ac-MVNAL were compared against melting temperatures without CoA-Ac-MVNAL with a t test (#### = *p*-value ≤ 0.0001). All measurements were performed in triplicates and are presented as means ± SD.

Having seen that the N- and C-termini impact on the enzymatic activity of *Ct*Naa50, we wanted to test whether they also influence thermostability using nano differential scanning fluorimetry (nanoDSF) (Figure 4). As MVNALE was the best substrate in our activity assays and the bisubstrate analog CoA-Ac-MVNAL has been characterized in previous *At*Naa50 and *At*Naa60 studies [21,39], we studied its stabilizing effect on *Ct*Naa50.

Without a ligand, the full-length *Ct*Naa50 expressed in *C. thermophilum* and the C-terminal deletion construct *Ct*Naa50_1-289_ expressed in *S. cerevisiae* had the highest melting temperatures (T_m_) of 61.6 ± 0.1 °C and 62.3 ± 0.6 °C, respectively. The N-terminal deletion construct *Ct*Naa50_82-445_ and the GNAT domain construct *Ct*Naa50_82-289_ were less stable, with T_m_ of 57.9 ± 0.1 °C and 57.0 ± 0.0 °C, respectively. When incubated with tenfold molar equivalents of CoA-Ac-MVNAL, all constructs were stabilized by more than 10 °C (73.2 ± 0.2 °C, 72.5 ± 0.2 °C, 68.6 ± 0.2 °C, and 71.2 ± 0.2 °C, respectively), indicating that they all bind CoA-Ac-MVNAL and are stabilized upon binding. These data show that *Ct*Naa50 is stabilized by its N-terminus, with *Ct*Naa50 and *Ct*Naa50_1-289_ having almost identical melting temperatures, which are at least 4 °C higher than for the *Ct*Naa50_82-445_ and *Ct*Naa50_82-289_ truncation variants. The C-terminus has no significant effect on thermostability.

In nanoDSF assays, the change in tryptophan fluorescence between the folded and unfolded protein conformation were measured. The first 81 residues of *Ct*Naa50 do not contain tryptophan residues, but the C-terminal extension features one tryptophan residue (full-length *Ct*Naa50 contains five tryptophan residues in total). Because there was no change in the observed melting temperature in the presence of the C-terminus, it is likely already unfolded, and therefore only the unfolding of the GNAT domain was detected.

### 2.5. Crystal Structure of CtNaa50 with a Bisubstrate Analog 

**Figure 5:**
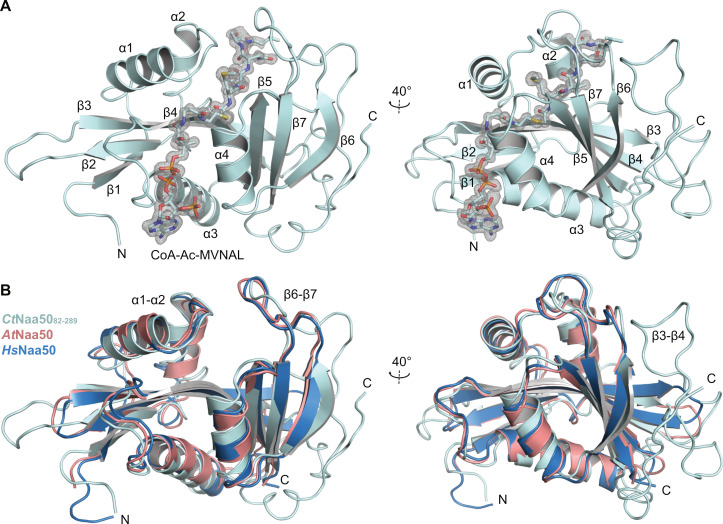
Structure of *Ct*Naa50_82-289_ in complex with CoA-Ac-MVNAL. (**A**) The structure of *Ct*Naa50_82-289_ (teal) in ribbon representation is shown in complex with CoA-Ac-MVNAL in sticks. The protein adopts a GNAT fold. The 2m*F_obs_-DF_calc_* maps are at a contour level of 1 σ. (**B**) *Ct*Naa50_82-289_ superimposes well with *At*Naa50/CoA-Ac-MVNAL (salmon) [39] and *Hs*Naa50/CoA/MLGPE (blue) [51]. Some loops are longer in *Ct*Naa50, especially loop β3–β4.

In order to characterize the *Ct*Naa50 GNAT domain, we crystallized *Ct*Naa50_82-289_ in complex with the bisubstrate analog (peptide sequence: M1_p_V2_p_N3_p_A4_p_L5_p_) and solved its X-ray crystal structure. Crystals of *Ct*Naa50_82-289_/CoA-Ac-MVNAL belong to space group P2_1_2_1_2_1_ with one molecule per asymmetric unit and diffracted to 1.1 Å resolution. The structure was determined by molecular replacement with the human Naa50/AcCoA structure (PDB: 2OB0) as a search model (Table 2). *Ct*Naa50_82-289_ shares the GCN5-related N-acetyltransferase (GNAT) fold with a mixture of four α helices and seven β strands (Figure 5A). The entire protein could be built from residues L82 to V289 with an additional glycine at position 81 as an expression artifact and five histidine residues at positions 290-294 from the hexa-histidine tag used for purification. In addition to the bisubstrate analog CoA-Ac-MVNAL, we also modeled one hexaethylene glycol molecule resulting from the crystallization condition and five glycerol molecules from the cryoprotectant into the density. *Ct*Naa50_82-289_/CoA-Ac-MVNAL superimposes well with other Naa50 structures, indicating a high degree of conservation (Figure 5B): root-mean-square deviations (RMSD) of 1.70 Å (for 151 Cα residues) with *Hs*Naa50/CoA/MLGPE (PDB: 3TFY, [51]), 1.92 Å (for 148 Cα residues) with *At*Naa50/CoA-Ac-MVNAL (PDB: 6Z00, [39]), and 1.79 Å (for 147 Cα residues) with *Sc*Naa50 in the *Sc*NatE complex (PDB: 4XNH). The bisubstrate analog CoA-Ac-MVNAL is perfectly resolved in the electron density. The conserved AcCoA-binding motif Q/RxxGxG/A in turn β4 to α3 comprises residues RSLGLA in *Ct*Naa50 and interacts with the pyrophosphate part of CoA. In addition to this turn, additional major binding sites for the CoA moiety are in α4 and β4.

All secondary structure elements are the same as in the yeast, human, and Arabidopsis structures. The major differences are in three loop regions β2–β3, β3–β4, and α3–β5, which are longer than in orthologous Naa50 proteins. In particular, loop β3–β4 has a length of 32 residues, which is 30 residues longer than the human protein (Figure 5A). This loop could be completely built and due to crystal packing folds back to the protein with the Q175 side-chain pointing towards the substrate peptide entry site. The amide group of Q175 (side-chain) is at 3.2 Å distance from the bisubstrate analog L5_p_ side-chain.

**Figure 6:**
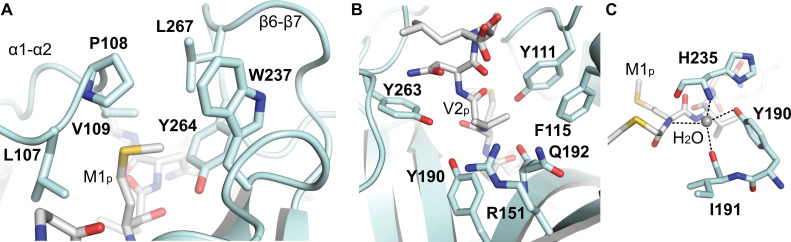
*Ct*Naa50_82-289_ active site and substrate peptide binding. (**A**) M1_p_ resides in a hydrophobic pocket formed by residues in loops α1–α2 (L107, P108, and V109), β6–β7 (Y264 and L267), and β5 (W237). (**B**) The second peptide residue, V2_p_, sits in an amphiphilic pocket formed by Y111, F115, R151, Y190, Q192, and Y263. (**C**) The active site contains a catalytically important water molecule, which is bound by the amide group of M1_p_, Y190 hydroxyl, I191 main-chain carbonyl, and H235 main-chain amide groups. Structural details of the active site are shown in the ribbon representation in light blue with residues shown in stick representation.

The peptide moiety of CoA-Ac-MVNAL binds at the substrate peptide binding site, while the two Naa50 loops, α1–α2 and β6–β7, connected by van der Waals contacts, fold over the peptide. Several residues surround M1_p_ to create a hydrophobic binding pocket, which is typical for active Naa50 enzymes: L107, P108, and V109 in loop α1–α2, and Y264 and L267 in loop β6–β7 as well as W237 in β5 (Figure 6A). Additionally, M1_p_ amide nitrogen forms a hydrogen bond with the β5 H235 carbonyl group, which is part of the active site.

The Q192 main-chain carbonyl group in β4 and the Y111 hydroxyl group in loop α1–α2 form hydrogen bonds with the substrate V2_p_ main-chain amide and carboxyl groups, respectively. V2_p_ is partly solvent-exposed and resides in an amphiphilic pocket formed by Y111 (loop α1–α2), F115 (α2), R151 (β3), Y190, Q192 (both β4), and Y263 (loop β6–β7) (Figure 6B). The surrounding of V2_p_ is positively charged and partially accessible to the solvent (Supplementary Figure S4). The side-chain of N3_p_ forms hydrogen bonds to main-chain carbonyl Y263 and Q175 and an intramolecular hydrogen bond to the main-chain amino group of L5_p_. A4_p_ and L5_p_ are placed at the entrance of the binding pocket and do not form sequence-specific intermolecular interactions. Interestingly and as mentioned above, the extended loop β3–β4 comes in close proximity to the fifth residue of the substrate peptide. Due to the hydrophobic substrate pocket, peptides starting with methionine followed by a hydrophobic residue at position 2 are ideal *Ct*Naa50 substrates.

The active site of *Ct*Naa50_82-289_ includes a tightly bound water molecule, which is important for acetylation and is also present in other active Naa50 structures [39,51]. The water is coordinated by four hydrogen bonds: M1_p_ main-chain amide group, Y190 side-chain hydroxyl group, I191 main-chain carbonyl group, and H235 main-chain amide group (Figure 6C). The interacting residues are conserved in active Naa50 proteins (Arabidopsis Y77, I78, H115 and human Y73, I74, H112) but not in the catalytically inactive yeast Naa50 (residues Q92, I93, and Y130). The *Ct*Naa50_82-289_ Y190F mutant showed reduced acetylation efficiency, which might be explained by impaired binding of the catalytic water molecule due to the missing hydroxyl group or a missing general acid, which would be required to protonate the leaving CoA molecule.

### 2.6. C. thermophilum Naa50 Does Not Bind to NatA

Naa50 interacts with the NatA complex to form a NatE complex in budding and fission yeast, humans, and Drosophila [34,44,45,46,62]. In *A. thaliana*, NatE complex formation is still under debate but might show similar dynamics as described in humans, where Naa50 exists alone as well as in complex with NatA [39,44,49]. As *Ct*Naa50 deviates by terminal elongations, we wanted to assess whether it also deviates with respect to NatA binding.

**Figure 7:**
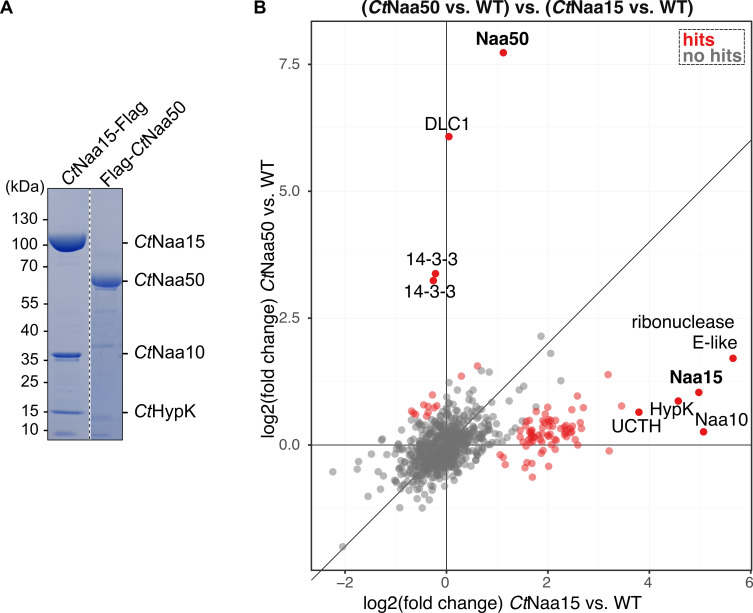
*C. thermophilum* pull-outs indicate that *Ct*Naa50 does not bind to *Ct*NatA. (**A**) *Ct*Naa50 does not copurify with NatA subunits in a pull-out, while Naa15 copurifies with Naa10 and HypK. SDS-PAGE gel was stained with Coomassie. (**B**) Mass spectrometry experiments confirm that *Ct*Naa50 and *Ct*Naa15 pull-outs do not lead to enrichment (red data points) of the respective other protein, highlighting that they do not interact. Putative dynein light chain 1 protein (DLC1) and two hypothetical 14-3-3 proteins were copurified with Naa50. A ribonuclease E-like protein, a ubiquitin carboxyl-terminal hydrolase-like protein (UCTH), and the NatA subunits Naa10 and HypK were enriched the most during Naa15 pull-out. TMT LC-MS/MS experiments were performed in triplicates.

We tagged Naa15 or Naa50 with a protein A-tag and expressed the proteins under the native actin promoter in *C. thermophilum*. The tagged proteins were extracted using IgG-Sepharose beads (Figure 7A), and copurified proteins from these pull-outs were identified and quantified by mass spectrometry using identifiable tandem mass tags (TMT LC-MS/MS) for each condition in triplicates (Figure 7B). Wild-type *C. thermophilum* cleared lysates were mock-purified on IgG-Sepharose beads as a reference (background) for each pull-out experiment. From the 748 identified proteins (Supplementary Data S1), we could identify enriched Naa50 and the NatA subunits Naa10 (NCBI identifier CTHT_0063490), Naa15 (CTHT_0031530), and HypK (CTHT_0058830) (Figure 7B, Supplementary Figure S5). Pull-outs using Naa50 as bait versus the untagged control resulted in the enrichment of Naa50, putative dynein light chain 1 protein (CTHT_0058240), and two hypothetical 14-3-3 proteins (CTHT_0054160 and CTHT_0054410) (Figure 7B, Supplementary Figure S5). In pull-outs with the tagged Naa15 protein versus the untagged control, we detected Naa10, Naa15, HypK, ubiquitin carboxyl-terminal hydrolase-like protein (CTHT_0063590), and an uncharacterized protein (CTHT_0074400; ribonuclease E-like) (Figure 7B, Supplementary Figure S5). Interestingly, Naa50 and Naa15 (NatA) were exclusively found in their respective pull-outs, suggesting that *Ct*Naa50 does not bind to NatA.

**Figure 8:**
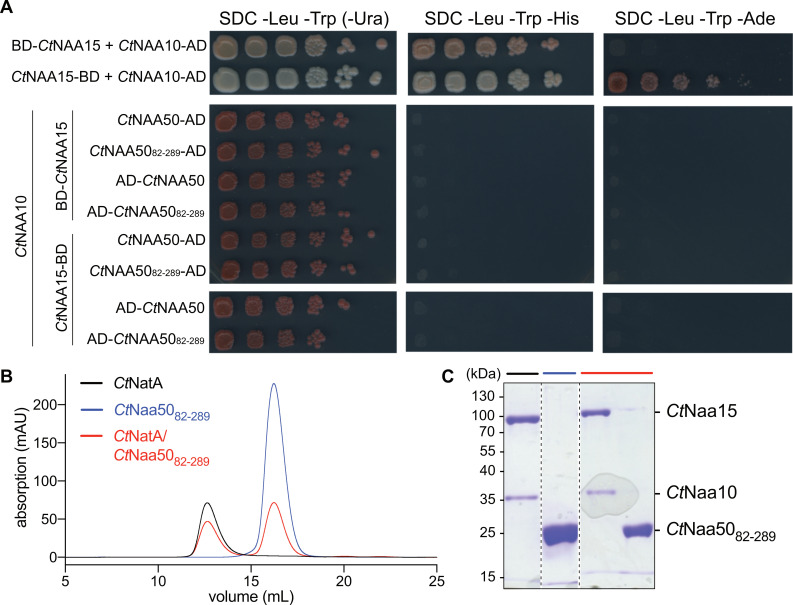
Analysis of *Ct*NatA/*Ct*Naa50 interaction using yeast two-hybrid assays and in vitro interaction studies. (**A**) Only *Ct*Naa15 and *Ct*Naa10 subunits interacted with each other in yeast two-hybrid assays. *Ct*Naa15 and *Ct*Naa50 constructs did not interact. Proteins were fused to Gal4 activation (AD) or binding domains (BD). Growth on SDC -Leu/-Trp indicates successful transformation. (**B**) *Ct*Naa50_82-289_ incubated with *Ct*NatA did not coelute on an analytical size-exclusion column (Superdex 200 10/300GL). (**C**) Peak fractions in (**B**) are visualized by SDS-PAGE stained with Coomassie.

The absence of an interaction between Naa50 and Naa15 (NatE formation) was further confirmed by yeast two-hybrid assays (Figure 8A). We fused *Ct*Naa15 to N- or C-terminal Gal4-binding domains (BD) and *Ct*Naa50 or *Ct*Naa10 proteins to N- or C-terminal Gal4 activation domains (AD). As expected, *Ct*Naa15 interacted strongly with *Ct*Naa10 in the positive control, and neither were self-activating (Supplementary Figure S6). In contrast, *Ct*Naa15 did not interact with full-length *Ct*Naa50 or *Ct*Naa50_82-289_. In order to stabilize Naa15, we additionally expressed Naa10 in yeast. We tried all possible Gal4 domain positions for Naa15 and Naa50 but could not observe any interaction.

In addition, we tested whether *Ct*Naa50_82-289_ and *Ct*NatA can bind in vitro using analytical size exclusion chromatography (Figure 8B,C). Again, no interaction was observed, as there was no shift of the elution peak for *Ct*NatA when incubated with *Ct*Naa50_82-289_.

The interaction between Naa15 and Naa50 is conserved in yeast and humans, with Naa50 loops β2–β3 and β4–α3 forming the major interacting sites [45,52]. While the latter loop has the same length in the orthologous proteins and shares conserved residues, loop β2–β3 is six residues longer in *C. thermophilum* and *N. crassa*. Double mutants in this loop from *Sp*Naa50 Y49A/V53K and *Hs*Naa50 Y50A/I54K showed reduced binding to the corresponding NatA complex. In *C. thermophilum* or *N. crassa,* the tyrosine is not conserved (L133 or L142, respectively), and the valine or isoleucine are replaced by lysine residues (K143 or K152, respectively). The superimpositions of the *Ct*Naa50_82-289_ structure on *Sc*Naa50 and *Ct*NatA (PDB identifier 5NNP) [29] on *Sc*NatA in the *Sc*NatE complex (PDB identifier 4XNH) indicate that the longer loop, β2–β3, would clash with *Ct*NatA near a Naa10–Naa15 interface if Naa50 would bind in the same manner (Supplementary Figure S7A). To test whether binding could simply be restored by shortening the β2–β3 loop of *Ct*Naa50_82-289_, we created two *Ct*Naa50 mutants: Δloop_137-142_, where the six additional residues were deleted, and Δloop_137-142/K143I/V144P_, which contains two additional mutations to better match the *Sc*Naa50 sequence. Both *Ct*Naa50_82-289_ Δloop mutants did not bind to *Ct*NatA (Supplementary Figure S7B,C). NatE complex formation probably involves more than this Naa50 loop region and is not conserved in these filamentous fungi.

Taken together, our pull-out experiments and the in vivo and in vitro binding assays show that *Ct*Naa50 does not bind to *Ct*NatA. 

### 2.7. C. thermophilum Naa50 Binds to Dynein Light Chain 1 Protein 

While our pull-out studies did not show a *Ct*Naa50/NatA interaction, putative dynein light chain 1 protein (DLC1) (NCBI identifier CTHT_0058240) was the second most enriched protein detected by mass spectrometry. Dynein light chain proteins typically bind to the elongated N-termini of the two dynein intermediate chains (DIC) as part of the dynein transport machinery [63]. Three light chain families (DLC) exist in humans (Roadblock, LC8, and Tctex), which all bind to different motifs as dimers. They stabilize the N-termini of DICs and may be involved in cargo recruitment of, for example, viruses [64]. However, numerous non-cargo-binding partners of DLC proteins outside of the dynein complex are known, e.g., p53-binding protein 1, Bim at the mitochondrial membrane, and large myelin-associated glycoprotein (L-MAG) [65,66,67].

We identified a known DLC1 binding motif, _34_RATQT_38_ (canonical (K/R)xTQT [68]), within the N-terminus of *Ct*Naa50. Surprisingly, the motif is weakly conserved within fungal Naa50 proteins. For example, in *C. globosum* the aligned region _34_NVATQT_39_ still features xTQT, while in *N. crassa* the aligned region is less conserved.

**Figure 9:**
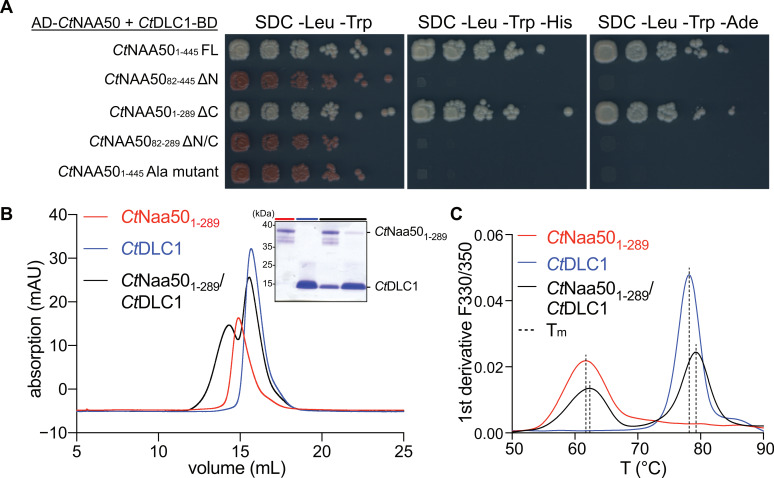
*Ct*Naa50 binds to *Ct*DLC1 with its N-terminal RATQT motif. (**A**) *Ct*NAA50 full-length (FL) interacted with *Ct*DLC1 in a yeast two-hybrid assay. Naa50 truncation variants lacking the N-terminus (ΔN or ΔN/C) and a poly-Ala mutant (DLC1 binding motif _34_RATQT_38_ mutated to _34_AAAAA_38_) did not interact with *Ct*DLC1. Coding sequences were fused to Gal4 activation (AD) or binding domains (BD). Growth on SDC -Leu/-Trp indicated successful transformation. (**B**) *Ct*Naa50_1-289_ incubated with *Ct*DLC1 led to an elution peak shift on an analytical size-exclusion column. Peak fractions are visualized by SDS-PAGE with Coomassie stain. (**C**) *Ct*Naa50_1-289_ and *Ct*DLC1 did not significantly differ in melting temperatures T_m_ when tested alone or in complex.

The interaction between *Ct*Naa50 and *Ct*DLC1 was confirmed by yeast two-hybrid and analytical SEC experiments. N-terminal truncation or mutating _34_RATQTT_39_ to a poly-Ala variant completely abolished the interaction in yeast two-hybrid assays (Figure 9A, Supplementary Figure S8A). Colonies with *Ct*Naa50 constructs containing the N-terminus and *Ct*DLC1 grew on strong selection media. Interestingly, *Nc*Naa50 did not interact with *Ct*DLC1 (Supplementary Figure S8B). In addition, incubating an excess of *Ct*DLC1 with *Ct*Naa50_1-289_ resulted in a peak shift of *Ct*Naa50_1-289_ on an analytical SEC (Figure 9B).

We tested whether the binding of *Ct*DLC1 influences the acetylation activity or thermostability of *Ct*Naa50_1-289_. The purified *Ct*Naa50_1-289_/*Ct*DLC1 complex acetylated the MVNALE peptide with a K_m_ of 30.4 ± 6.3 µM and a k_cat_ of 21.4 ± 1.2 min^−1^ (Supplementary Figure S2E). In comparison, *Ct*Naa50_1-289_ alone had almost identical Michaelis-Menten parameters, with a slightly lower k_cat_ (K_m_ = 31.9 ± 4.4 µM and k_cat_ = 16.3 ± 0.6 min^−1^). *Ct*DLC1 did not increase the thermostability of *Ct*Naa50_1-289_. Both proteins unfolded at their respective melting temperatures when in complex (Figure 9C). *Ct*Naa50_1-289_ unfolded at 62.1 ± 0.1 °C (alone at 61.7 ± 0.0 °C) and *Ct*DLC1 at 79.2 ± 0.0 °C (alone at 78.1 ± 0.1 °C). This indicates that complex formation does not impact on protein stability under in vitro conditions. 

### 2.8. Naa50 Binds to the Ribosome with Its C-Terminus

The NatE complex in humans and yeast acts cotranslationally [7]. The yeast cryo-EM structure of the ribosome/NatE complex revealed that Naa50 interacts with ES7a, while Naa15 binds to the rRNA helices H24 and H47 and to ES27a and ES39a [53]. As we could not detect an interaction of the elongated fungal Naa50 with NatA, we tested if Naa50 alone can bind to the ribosome. We identified two positive patches in the C-terminal extension, a highly conserved _431_KKRKGR_436_ motif (*C. thermophilum*; e.g., _480_KKKKGR_485_ in *N. crassa*) and the _394_RGRR_397_ region (present in *C. thermophilum*). 

**Figure 10:**
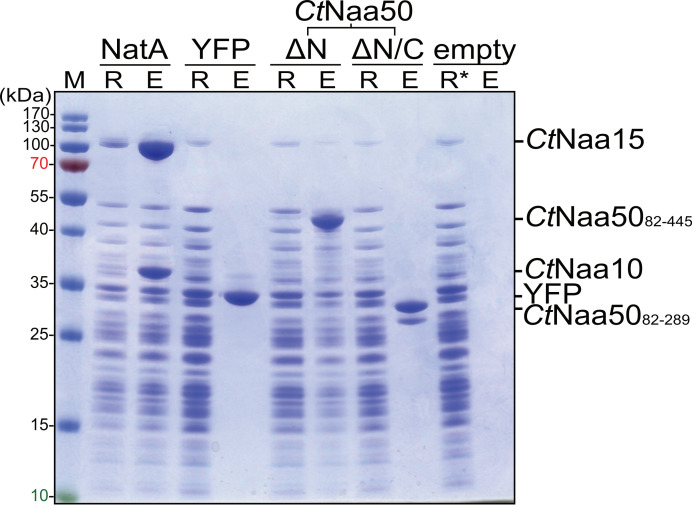
*Ct*Naa50 coelutes with *Ct*80S ribosomes via its C-terminus. In a Flag-tag pull-down experiment, Flag-tagged proteins were eluted (E) from Flag beads after incubation with *Ct*80S ribosomes (R; ribosome flow-through). The negative controls YFP and empty beads do not bind ribosomes. *Ct*NatA and *Ct*Naa50_82-445_ (ΔN) coelute with ribosomes, while *Ct*Naa50_82-289_ (ΔN/C) does not bind to the ribosome. M = Protein size marker; * highlights a pure ribosomal fraction.

To test whether the elongated Naa50 could bind to *Ct*80S ribosomes, we used a Flag-tag pull-down assay. *Ct*NatA served as positive control, and YFP and empty Flag beads served as negative controls. The *Ct*Naa50_82-445_ variant containing the C-terminus with the two positive patches bound to the ribosome, while the *Ct*Naa50_82-289_ GNAT domain was not able to bind (Figure 10). Naa50 binding alone, without NatA, to the ribosome had not been observed before. It might allow Naa50 to acetylate substrates cotranslationally without its typical binding partner, NatA. Such cotranslational Naa50 activity and its in vivo relevance have to be analyzed in detail. 

## 3. Discussion

**Figure 11:**
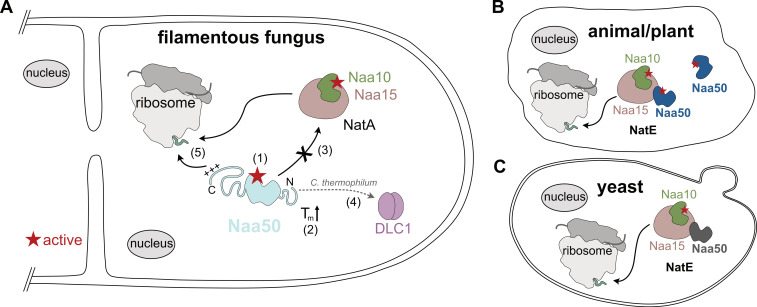
Diversity of Naa50 in different organisms. (**A**) Naa50 (teal) from filamentous fungi contains significant N- and C-terminal extensions and shows N-terminal acetylation activity towards typical NatC/E/F-type substrates (1). The N-terminus increases its thermostability (2). It does not interact with NatA, and therefore a NatE complex is not formed in filamentous fungi (3). In *C. thermophilum*, a binding motif within the N-terminus allows for interaction with DLC1 (4). Positive patches within the C-terminus enable ribosome binding without NatA (5). (**B**) Naa50 in animals and plants (blue) is active and can form a NatE complex with NatA. (**C**) Yeast Naa50 (grey) is inactive due to missing catalytic residues. It forms a NatE complex and can bind to the ribosome as NatE.

Naa50 is among the functionally and structurally best described Nα acetyltransferases [6]. Strikingly, it shows remarkable species-specific diversity with respect to complex formation and activity (Figure 11). With this study, we add important aspects to this diversity, as filamentous fungi *Chaetomium thermophilum* and *Neurospora crassa* feature Naa50 orthologs with significant N- and C-terminal extensions (Figure 11A). Interestingly, these Naa50 proteins contain 67 (15.1%; *Ct*Naa50) or 65 proline residues (13.2%; *Nc*Naa50), which are especially enriched at the N-terminus. For comparison, human Naa50 only contains five (3%) proline residues. As a high proline count is a characteristic of thermophilic organisms [69], this could explain the observed thermostability of *Ct*Naa50 and, in particular, the observed impact of the N-terminus. 

We investigated the phenotypic effect of knocking out Naa50 in *N. crassa*. Compared to *naa10*, *naa15*/wt, and *hypK* knockout strains, *naa50* had a mild phenotypic growth defect with less conidia formation and faster growth than the wild type. Similarly, NAA50 knockout in Arabidopsis led to small plants with only few leaves [48], and knockout in human cells or Drosophila led to improper chromatid separation [44,46,47]. On the other hand, knocking out the inactive yeast Naa50 did not show any phenotype [34]. The depletion of NAA10 or NAA15 in *Arabidopsis thaliana* also resulted in a growth defect, while NAA10 knockout was lethal [70]. NatA (Naa10 and Naa15) is responsible for acetylating 38% of the human proteome, while NatC/E/F together acetylate 21% [2]. Knocking out components of NatA (NAA10/NAA15) likely affects organisms more severely than knocking out NAA50 due to the partly overlapping substrate specificity of NatC/E/F.

The excessive N- and C-terminal extensions have no severe impact on acetylation activity in vitro when tested with C-terminal (*Ct*Naa50_1-289_) or N-terminal (*Ct*Naa50_82-445_) deletions and with the GNAT domain (*Ct*Naa50_82-289_). In comparison, full-length *Ct*Naa50 purified from *C. thermophilum* showed lower end-point acetylation in vitro. It may contain species-specific modifications, which could affect its activity. It is noteworthy that *Ct*Naa50 from pull-outs ran at a slightly higher molecular weight, as expected on SDS-PAGE gels. Additionally, the expression and purification of full-length *Ct*Naa50 in different organisms was unsuccessful. *Ct*Naa50_82-289_ and *Nc*Naa50_93-287_ had almost identical acetylation efficiencies (k_cat_/K_m_), while *Ct*Naa50_82-289_ showed a higher affinity for AcCoA (K_m_) and *Nc*Naa50_93-287_ acetylated faster (k_cat_). All acetylation assays were performed at 30 °C, which may be disadvantageous for *Ct*Naa50, which has an optimal growth temperature around 50–55 °C [71].

As *Nc*Naa50_93-287_ and *Ct*Naa50_82-289_ both share the characteristic substrate specificity and activity of orthologous Naa50 proteins, we solved the structure of *Ct*Naa50_82-289_ with a bisubstrate analog CoA-Ac-MVNAL and compared it to other Naa50 structures. *Hs*Naa50/CoA/MLGPE peptide (PDB: 3TFY, [51]), *At*Naa50/CoA-Ac-MVNAL (PDB: 6Z00, [39]), and *Sc*Naa50 in the *Sc*NatE complex (PDB: 4XNH) superimpose very well with *Ct*Naa50_82-289_/CoA-Ac-MVNAL. It shares typical structural features of active Naa50, namely, a hydrophobic binding pocket for methionine substrates and histidine and tyrosine residues as well as a coordinated water molecule in the active site. The GNAT domain *Ct*Naa50_82-289_ contains the elongated loops β2–β3, β3–β4, and α3–β5 but overall closely resembles known Naa50 structures. 

Naa50 can associate with NatA to form the NatE complex in yeast, Drosophila, and humans, while in Arabidopsis Naa50 colocalizes with Naa10 (Figure 11B,C) [34,44,46,50]. Interestingly, more than 80% of Naa50 does not interact with NatA in HeLa cells, while Naa50 was exclusively complexed to NatA in pull-out experiments with Drosophila and yeast [34,44,46]. The elongated *C. thermophilum* Naa50, however, did not bind to Naa15 (NatA), as analyzed by pull-out experiments and yeast two-hybrid and analytical SEC assays. Naa15 proteins from yeast and humans contain a central conserved TPTLxE motif, which is important for Naa50 binding. Mutating the second threonine (*S. pombe* T412, *S. cerevisiae* T416, or human T406) to tyrosine abolished the NatA–Naa50 interaction [45]. The corresponding residues of *Ct*Naa15 or *Nc*Naa15 are DPKNVD (residues 403-408 for *Ct*Naa15 or 399-408 for *Nc*Naa15, respectively), and therefore the interaction motif is not conserved. Taken together, this shows that NatE is not formed in these fungi. 

While Naa50 does not interact with NatA, dynein light chain 1 protein was identified by an MS analysis as a binding partner of *C. thermophilum* Naa50. With 69% sequence identity, the identified *Ct*DLC1 is a homolog of the human dynein light chain 1 (DYNLL1 or LC8). Interestingly, only *Ct*Naa50 contains the binding motif _34_RATQT_38_ (canonical (K/R)xTQT [68]) but not *Nc*Naa50. We only discovered *Ct*DLC1 in the pull-out experiments and no other dynein subunits, which implies that *Ct*Naa50 is unlikely to be recruited to the dynein motor machinery. The major function of DLC1 is to serve as a dimerization platform [72]. Because the small protein forms a homodimer that contains two binding sites for interacting proteins, it brings binding partners to close contact, resulting in a heterotetrameric complex. This, for example, has an anti-apoptotic effect in the case of Bim as its binding partner [67]. Interestingly, the intrinsically disordered binding regions of DLC1 partners become more ordered upon the dimerization and binding of DLC1, which could prevent ubiquitin-independent 20S proteasomal degradation [67,72]. *Ct*DLC1 does not affect the acetylation activity of *Ct*Naa50_1-289_ in vitro. In the case of *Ct*Naa50, the binding of *Ct*DLC1 may also protect Naa50. The N-terminus of *Ct*Naa50 increases its thermostability, and *Ct*DLC1 binding may further stabilize the protein in vivo. As *C. thermophilum* has a higher optimal growth temperature (50–55 °C, [71]) than *N. crassa* (25 °C, [73]), this additional interaction may contribute to its in vivo stability.

As *Ct*Naa50 does not interact with NatA, we investigated whether it could bind to the ribosome on its own. Therefore, *Ct*Naa50_82-445_ was tested for ribosome binding using a Flag-tag pull-down assay. *Ct*80S ribosomes coeluted with *Ct*Naa50_82-445_, similar to *Ct*NatA (positive control). In contrast, *Ct*Naa50_82-289_, lacking the C-terminal extension, was not able to copurify with ribosomes. These data indicate that Naa50 alone indeed binds to 80S ribosomes and that the C-terminal extension in filamentous fungi is crucial for binding. So far, Naa40 (NatD) is the only NAT that has been suggested to bind to the ribosome on its own without an adaptor [74,75]. In yeast, the majority of NatE ribosome binding results from positive patches on Naa15, while Naa50 has only a small interface with ES7a [53,76]. *Ct*Naa50 contains two positively charged regions close to its C-terminus, a not conserved _394_RGRR_397_ patch and a highly conserved _431_KKRKGR_436_ motif (*C. thermophilum*; _480_KKKKGR_485_ in *N. crassa*). Similar positively charged patches have been shown to be important for the binding of other ribosome-associated factors, including targeting factors such as chloroplast SRP54 (RRKRK) [77], and chaperones, including yeast βNAC (RRKx_n_KK) [78] and SSB (KK and KR pairs) [79]. The ribosome binding of Naa50 alone seems to follow the same principles as employed by other ribosome-associated factors and might enable cotranslational function without an additional adaptor subunit.

Taken together, our data highlight the importance of the Naa50 N-and C-terminal extensions found in fungal proteins (Figure 11). While the N-terminal extension increases *Ct*Naa50 thermostability and contributes to the recruitment of a newly identified partner, the C-terminal extension contains conserved interaction motifs for ribosome binding. Strikingly, *Ct*Naa50 does not associate with NatA, and the ribosome binding of Naa50 alone might allow for cotranslational acetylation. Overall, our data contribute to the diversity of Nα acetyltransferases, with Naa50 showing striking species-specific differences that require further studies.

## 4. Materials and Methods

### 4.1. Neurospora crassa Race Tube Growth Phenotypes

We used the standard *Neurospora crassa* wild-type strain 74-OR23-1VA (FGSC 2498, *matA*) and the mutant strains FGSC #18489 (NCU08944, *naa10*), FGSC #23473-heterokaryon (NCU07936, *naa15*), FGSC #19718 (NCU00576, *naa50*), and FGSC #13843 (NCU02784, *hypK*). The strains were obtained from the Fungal Genetics Stock Center (FGSC, Manhattan, KS, USA, http://www.fgsc.net) [80]. All strains were maintained by growth in Vogel’s minimal medium with 1.5% sucrose as the carbon source. Strain manipulation and growth medium preparation followed standard procedures and protocols [81]. See also the *Neurospora* protocol guide (http://www.fgsc.net/Neurospora/NeurosporaProtocolGuide.htm).

*N. crassa* strain race tube assays were performed as previously described [82] in medium consisting of 1× Vogel’s salts, 0.1% glucose, 0.17% arginine, 50 ng mL^−1^ biotin, and 1.5% bacto agar.

### 4.2. Plasmid Construction

For expression in *Escherichia coli*, *Ct*NAA15 and *Ct*NAA10 (*Ct*NatA complex) were cloned as described previously [29]. The full-length *Ct*NAA50-coding sequence was PCR-amplified from *Chaetomium thermophilum* cDNA. The GNAT-domain-coding sequence of *Ct*NAA50 (*Ct*Naa50_82-289_) was subsequently PCR-amplified with an additional C-terminal hexa-histidine tag and digested with NcoI and BamHI restriction enzymes. The fragment was ligated into the multiple cloning site of pET24d (Merck—Novagen, Darmstadt, Germany). *Ct*Naa50_82-289_ Y190F, H235A, and Δloop mutants were generated using QuikChange Lightning site-directed mutagenesis (Agilent Technologies Germany GmbH & Co. KG, Waldbronn, Germany). The GNAT-domain-coding sequence of *Nc*NAA50 (*Nc*Naa50_93-287_) was PCR-amplified from *Neurospora crassa* cDNA with an additional C-terminal hexa-histidine tag. The amplicon was NcoI/BamHI digested and ligated into the multiple cloning site of pET24d (Merck—Novagen, Darmstadt, Germany). The *Ct*DLC1-coding sequence was ordered from Integrated DNA Technologies (Coralville, Iowa, USA) and cloned with or without a C-terminal hexa-histidine tag into pET24d (Merck—Novagen, Darmstadt, Germany) using NcoI/BamHI digestion and ligation. For Flag-tag pull-down assays, coding sequences were PCR-amplified to generate different tags: *Ct*NAA10 was cloned into pET21d with a C-terminal Flag-tag, *Ct*NAA15 with a TEV-cleavable N-terminal hexa-histidine and a C-terminal Strep2-tag, and *Ct*NAA50 (*Ct*Naa50_82-289_ and *Ct*Naa50_82-445_) and YFP each with TEV-cleavable N-terminal hexa-histidine-, N-terminal Flag-, and C-terminal Strep2-tags into pET24d.

For expression in *Chaetomium thermophilum*, full-length *Ct*NAA15- or *Ct*NAA50-coding sequences were PCR-amplified and cloned into pRSF-duet-FTpA or pRSF-duet-pAFT plasmids, respectively, using EcoRI/NotI digestion and ligation. *Ct*NAA15 and *Ct*NAA50 contained C- and N-terminal, respectively, protein A-tag (pA; TEV protease-cleavable) and Flag-tag (FT; uncleavable).

For expression in *Saccharomyces cerevisiae*, a *Ct*NAA50 C-terminal truncation (*Ct*Naa50_1-289_)-coding sequence was PCR-amplified with a TEV-cleavable N-terminal His-tag. The fragments were cloned (NcoI/BamHI digestion and ligation) into pMT929 (created by M. Thoms and modified by N. Dobrev, BZH: a 2 μ vector with a bidirectional GAL1/GAL10 promoter, resistance markers for TRP1, LEU2, and Amp, and a TEV-cleavable N-terminal histidine tag).

For yeast two-hybrid analyses, all constructs were cloned into respective plasmids [83,84] that were modified for NcoI/BamHI digestion and ligation. A full-length *Ct*NAA15-coding sequence was ligated into pG4BDN22 and pG4BDC22 to create Gal4-binding domain fusion constructs. A full-length *Ct*NAA10-coding sequence was cloned into pG4ADC111 for a C-terminal Gal4 activation domain fusion. *Ct*NAA50- and *Nc*NAA50-variant-coding sequences were cloned into pG4ADHAN111 and pG4ADC111 to generate Gal4 activation domain fusion constructs. _34_RATQT_38_ to _34_AAAAA_38_ mutant pG4ADHAN111::*Ct*NAA50 was generated with QuikChange Lightning site-directed mutagenesis (Agilent Technologies Germany GmbH & Co. KG, Waldbronn, Germany). *Ct*DLC1 was cloned into pG4BDC22. Additionally, *Ct*NAA10 was cloned into a modified pRS426 plasmid with an ADH1 promoter and an N-terminal nuclear localization sequence (NLS) for the stabilization of *Ct*Naa15.

All primers are listed in Supplementary Table S1, and the generated plasmids are listed in Supplementary Table S2.

### 4.3. Expression in E. coli and Purification of Proteins

*Ct*Naa50_82-289_, *Ct*Naa50_82-445_, *Ct*DLC1, YFP, and the *Ct*NatA complex (*Ct*Naa10-His_6_/*Ct*Naa15) were expressed in Rosetta II (*DE3*) *E. coli* (Merck—Novagen, Darmstadt, Germany) in ZY autoinduction medium supplemented with 5052, trace elements, chloramphenicol (34 mg L^−1^), kanamycin (50 mg L^−1^), and ampicillin (100 mg L^−1^; in case of *Ct*NatA) [85] at 37 °C. Cells were cultivated from OD_600_ = 0.8–1.0 at 18 °C. *Ct*Naa50_82-289_ was purified as follows: Cells were harvested, resuspended in buffer A_500_ (20 mM pH 7.5 HEPES, 500 mM NaCl, 20 mM imidazole) supplemented with a protease inhibitor mix (SERVA Electrophoresis GmbH, Heidelberg, Germany), and lysed with a microfluidizer (M1-10L, Microfluidics, Westwood, MA, USA). The lysate was cleared for 30 min at 50,000× *g* at 4 °C and filtered through a 0.45 µm membrane. The supernatant was applied to a 1 mL HisTrap FF column (GE Healthcare, Chicago, IL, USA) for Ni-IMAC (immobilized metal affinity chromatography) purification. The column was washed with buffer A_500_, and the proteins were eluted with buffer A_500_ supplemented with 250 mM imidazole. *Ct*Naa50_82-289_ was subsequently purified by SEC (size-exclusion chromatography) using a Superdex 75 26/600 gel filtration column (GE Healthcare, Chicago, IL, USA) in buffer G_500_ (20 mM pH 7.5 HEPES, 500 mM NaCl). The *Ct*NatA complex was purified using the same method as *Ct*Naa50_82-289_ but with the buffers A_250_ (20 mM pH 8.0 HEPES, 250 mM NaCl, 40 mM imidazole) and G_250_ (20 mM pH 8.0 HEPES, 250 mM NaCl) and a Superdex 200 26/600 gel filtration column (GE Healthcare, Chicago, IL, USA). *Ct*DLC1-His_6_ was purified using the same method as *Ct*Naa50_82-289_ but with the buffers A_200_ (20 mM pH 7.5 HEPES, 200 mM NaCl, 20 mM imidazole) and G_200_ (20 mM pH 7.5 HEPES, 200 mM NaCl).

*Nc*Naa50_93-287_ was expressed in Rosetta II (*DE3*) *E. coli* (Merck—Novagen, Darmstadt, Germany) as before and purified in a two-step purification approach similar to *Ct*Naa50_82-289_. IMAC was performed with buffer A_gly_ (20 mM pH 8.5 HEPES, 250 mM NaCl, 5 % (*v*/*v*) glycerol, 20 mM imidazole) and A_gly_ supplemented with 250 mM imidazole for elution. *Nc*Naa50_93-287_ was finally purified on a Superdex 75 26/600 gel filtration column (GE Healthcare, Chicago, IL, USA) in the buffer G_gly_ (20 mM pH 8.5 HEPES, 250 mM NaCl, 5 % (*v*/*v*) glycerol). 

Flag-tagged *Ct*NatA, *Ct*Naa50 variants, and YFP were purified as before but with the following changes: Ni-IMAC was performed with buffer A_200_, and the hexa-histidine tag was removed by TEV digestion overnight in dialysis buffer at 4 °C (20 mM pH 7.5 HEPES, 200 mM NaCl, 15 mM imidazole, 5 mM β-mercaptoethanol). Tags and TEV protease were removed by reverse Ni-IMAC. Proteins were further purified on 5 mL StrepTrap HP columns (GE Healthcare, Chicago, IL, USA) with buffer A_200_, and proteins were eluted with 2.5 mM D-desthiobiotin. Proteins were finally purified with Superdex 200 16/600 gel filtration columns (GE Healthcare, Chicago, IL, USA) in buffer G_200_.

### 4.4. Expression in C. thermophilum and Purification of Proteins

The pRSF-duet-Pactin:pATF-*Ct*NAA50 and pRSF-duet-*Ct*NAA15-FTpA plasmids were used for ectopic integration and expression in *Chaetomium thermophilum*. The NAA50 open reading frame was amplified by PCR from *Chaetomium thermophilum* genomic DNA and fused to the actin promoter protA-TEV-Flag-tag, resulting in the pRSF-duet-Pactin:pATF-*Ct*NAA50 plasmid. The NAA15 promoter region (623 bases) and open reading frame were amplified by PCR from *Chaetomium thermophilum* genomic DNA and fused to the Flag-TEV-protA-tag, resulting in the pRSF-duet-*Ct*NAA15-FTpA plasmid. The *Chaetomium thermophilum* wild-type strain [86] was transformed with each plasmid as described in [87]. In brief, protoplasts were generated from the cell wall digestion of the fungus mycelium and mixed with the linearized plasmid DNA. The transformed protoplasts were plated and selected on CCM-sorbitol agar plates, supplemented with 0.5 mg/mL terbinafine, and incubated at 50 °C for two to three days. The expression of the pATF-NAA50 and NAA15-FTpA proteins was verified by Western blotting of whole-cell lysate using PAP (P1291, Sigma-Aldrich Chemie GmbH, Taufkirchen, Germany) antibodies according to the manufacturer’s protocol. The *C. thermophilum* strains used in this study are listed in Supplementary Table S3.

Mycelium was harvested and frozen in beads using liquid nitrogen. Mycelium beads were lysed in an MM400 cryogenic mixer mill (Retsch GmbH, Haan, Germany) at 30 s^−1^ for five minutes. The milled lysate was resuspended in lysis buffer (20 mM pH 7.5 HEPES, 500 mM NaCl, 20 mM imidazole, 0.1 % nonident P40) supplemented with a protease inhibitor mix (SERVA Electrophoresis GmbH, Heidelberg, Germany). The lysate was cleared for 30 min at 50,000× *g* at 4 °C and filtered through a 0.45 µm membrane. The supernatant was applied onto 600 µL of IgG Sepharose 6 FF resin (Cytiva Europe GmbH, Freiburg, Germany) for 14 h. The resin was washed several times with buffer C_500_ (20 mM pH 7.5 HEPES, 500 mM NaCl, 20 mM imidazole, 0.01% nonident P40), and the proteins were eluted through TEV protease cleavage at 4 °C for 4 h. For in vitro assays, *Ct*Naa50 was subsequently purified by SEC (size-exclusion chromatography) using a Superdex 200 10/300 GL gel filtration column (GE Healthcare, Chicago, IL, USA) in buffer G_500_ (20 mM pH 7.5 HEPES, 500 mM NaCl). 

### 4.5. Expression in S. cerevisiae and Purification of Proteins

*Ct*Naa50_1-289_ was expressed in the *Saccharomyces cerevisiae* strain DS1-2b (MATα; trp1-∆63; his3-∆200; ura3-52; leu2-∆1) under GAL promoter induction. After transformation, cells were selected on synthetic dextrose complete (SDC) medium lacking tryptophan. Positive clones were grown in SDC-Leu overnight at 30 °C and were used to inoculate YPG (yeast extract, peptone, galactose) medium. Cultures were grown at 30 °C for 20–24 h. Cells were harvested, resuspended in 10% glycerol, and flash-frozen in beads using liquid nitrogen.

Cell beads were lysed in an MM400 cryogenic mixer mill (Retsch GmbH, Haan, Germany) at 30 s^−1^ for three minutes. The milled lysate was resuspended in lysis buffer A_500_ (20 mM pH 7.5 HEPES, 500 mM NaCl, 20 mM imidazole) supplemented with a protease inhibitor mix (SERVA Electrophoresis GmbH, Heidelberg, Germany). The lysate was cleared for 30 min at 50,000× *g* at 4 °C and filtered through a 0.45 µm membrane. The supernatant was applied to 500 µL of Ni Sepharose 6 FF resin (Cytiva Europe GmbH, Freiburg, Germany) for Ni-IMAC (immobilized metal affinity chromatography) purification. The resin was washed with buffer A_500_, and the proteins were eluted through TEV protease cleavage at 4 °C for 4 h. Proteins were further purified with a Superdex 200 16/600 gel filtration column (GE Healthcare, Chicago, IL, USA) in buffer G_500_.

The *Ct*Naa50_1-289_/*Ct*DLC1 complex was purified similar to *Ct*Naa50_1-289_, but untagged *Ct*DLC1 *E. coli* lysate was mixed with the *S. cerevisiae* lysate prior to the IMAC step.

### 4.6. Yeast Two-Hybrid (Y2H) Assays

To test Naa15’s interactions with Naa10 or Naa50, the *Saccharomyces cerevisiae* strain PJ69–4A [88] was co-transformed systematically with pG4BDN22::*Ct*NAA15 or pG4BDC22::*Ct*NAA15 and with the pG4ADC111::*Ct*NAA10 and pG4ADHAN111::*Ct*NAA50 variants or the pG4ADC111::*Ct*NAA50 variants. In the case of the *Ct*NAA15 and *Ct*NAA50 assays, pRS426::*Ct*NAA10 was additionally co-transformed. To test Naa50’s interaction with dynein light chain 1 protein, the pG4ADHAN111::*Ct*NAA50 variants with pG4BDC22::*Ct*DLC1 as well as pG4ADC111::*Nc*NAA50 and pG4BDC22::*Nc*NAA50 were systematically tested with all *Ct*DLC1 plasmids. Double and triple transformants were selected on synthetic dextrose complete (SDC) medium lacking leucine, tryptophan, and optional uracil (SDC-Leu-Trp (-Ura)). For each transformation, four colonies were pooled and diluted to an OD_600_ of 3.0 in water, followed by ten-fold serial dilutions, and spotted onto SDC-Leu-Trp (-Ura), SDC-Leu-Trp-His, and SDC-Leu-Trp-Ade. Growth was recorded after 3 days of incubation at 30 °C. Transactivation controls were systematically performed for all positive constructs.

### 4.7. Analytical Size-Exclusion Chromatography

Experiments were performed in buffer G_500_ on a Superdex 200 10/300 GL gel filtration column (GE Healthcare, Chicago, IL, USA) with a 10 min incubation of the proteins prior to the runs. Elution peaks of *Ct*NatA complex (1.61 nmol), *Ct*Naa50_82-289_ (15.74 nmol), and a *Ct*NatA/*Ct*Naa50_82-289_ mixture (1.07/10.5 nmol) profiles were analyzed. For the shortened *Ct*Naa50_82-289_ loop β2–β3 variants, 8 nmol *Ct*Naa50_82-289_ Δloop_137-142_ or Δloop_137-142/K143I/V144P_ mutants were tested with and without 0.8 nmol *Ct*NatA. To analyze the binding of *Ct*DLC1 to *Ct*Naa50_1-289_, elution peaks of *Ct*DLC1 (24 nmol), *Ct*Naa50_1-289_ (4 nmol), and a mixture (24/4 nmol) were analyzed. The peak fractions were visualized by Coomassie-stained SDS-PAGE gels.

### 4.8. Crystallization of CtNaa50_82-289_/CoA-Ac-MVNAL

Crystallization was performed at 18 °C using the sitting drop vapor diffusion method. *Ct*Naa50_82-289_ was concentrated to 62 mg/mL and mixed in a 1:2 molar ratio with CoA-Ac-MVNAL. Crystals grew in 300 nL of protein and 300 nL of precipitant solution (0.1 M pH 6.0 MES, 8 % (*w*/*v*) PEG 3000, and 37 % (*v*/*v*) PEG 400) and appeared after 1–2 days. Crystals were cryoprotected with 20 % glycerol and flash-frozen in liquid nitrogen.

### 4.9. Data Collection, Structure Determination, and Analyses

The dataset was collected at beamline P13 (Deutsches Elektronen-Synchrotron DESY, Hamburg, Germany) at a cryogenic temperature. Images were integrated with XDS [89] and then scaled using AIMLESS [90] as part of the CCP4i software package [91,92]. Phases were obtained by molecular replacement with PHASER-MR [93], implemented in the PHENIX package [94]. The *Hs*Naa50/AcCoA structure (PDB: 2OB0) served as a search model for *Ct*Naa50_82-289_/CoA-Ac-MVNAL. Finally, iterative model building and refinement were performed with *Coot* [95] and Phenix.refine [96]. The resulting structural model quality was analyzed with MolProbity [97]. Structure figures were prepared with PyMOL (The PyMOL Molecular Graphics System, Schrödinger, LLC., New York City, NY, USA). Crystallographic data are summarized in Table 2. Coordinates and structure factors are deposited at the Protein Data Bank (PDB) with accession code 7OJU.

Multiple sequence alignments were performed using Clustal Omega (https://www.ebi.ac.uk/Tools/msa/clustalo/) [56] and were visualized with ESPript 3.0 (http://espript.ibcp.fr) [57]. The electrostatic surface potential of Naa50 was computed using the APBS-PDB2PCR plugin in PyMOL [98].

### 4.10. Colorimetric In Vitro Activity Assays

In vitro acetylation activity assays were performed using a microplate assay as described earlier [99] and as previously modified [39]. *Ct*Naa50 variant reactions were performed at 30 °C in triplicates (*n* = 3). For testing the substrate specificity, 1.5 mM peptides (MASS, MDEL, MLGTE, MVNALE, SESS; PLS GmbH, Heidelberg, Germany) were used with 500 mM AcCoA in 20 mL of DTNB buffer (10 mM 5,5‘dithiobis(2-nitrobenzoic acid), 100 mM pH 6.8 sodium phosphate dibasic, 10 mM EDTA) and 50 mL of reaction buffer (100 mM pH 7.5 HEPES, 100 mM NaCl, and 2 mM EDTA). Then, 500 nM (5 µM in the case of the Y190F and H235A mutants) enzymes in 50 mL were added to start the reaction. OD_412nm_ was recorded after 20 min and was converted to the produced CoA concentration. Control reactions without peptide were performed.

Michaelis–Menten kinetics were performed with 1.5 mM MVNALE peptide and increasing AcCoA concentrations (6.25–500 mM). OD_412nm_ was recorded after starting the reaction every 12 s, and the CoA concentrations were plotted against time. The initial slopes (velocity v) were plotted against the AcCoA concentration, and the Michaelis–Menten constant, K_m_, and maximum velocity, v_max_, were determined using GraphPad Prism (GraphPad Software, San Diego, CA, USA).

### 4.11. Nano Differential Scanning Fluorimetry (nanoDSF)

The melting temperatures of the *Ct*Naa50 variants with and without CoA-Ac-MVNAL were determined using nanoDSF in triplicates. First, 10 µM proteins in buffer G_500_ with or without 100 µM CoA-Ac-MVNAL were transferred into nanoDSF-grade glass capillaries (10 µL; NanoTemper Technologies GmbH, Munich, Germany) and measured with a Prometheus NT.48 (NanoTemper Technologies GmbH, Munich, Germany). A temperature gradient from 15–95 °C at 1.5 °C/min was applied. The PR.ThermControl software (NanoTemper Technologies GmbH, Munich, Germany) was used to calculate the T_m_ based on the F_330_/F_350_ ratio.

### 4.12. Statistical Analyses of In Vitro Acetylation and Melting Temperatures

Statistical analyses of triplicate (*n* = 3) results were performed using GraphPad Prism (GraphPad Software, San Diego, CA, USA). An ordinary one-way ANOVA test with Dunnett’s correction was used when comparing several datasets (for example, the acetylation of different peptides versus control). An unpaired t test was used when comparing two datasets (for example, melting temperatures with or without inhibitor). Statistical significance is represented as not significant (*ns*) or with one to four asterisks (* or # with *p*-value ≤ 0.0332, ** or ## with *p*-value ≤ 0.0021, *** or #### with *p*-value ≤ 0.0002, and **** or ##### with *p*-value ≤ 0.0001).

### 4.13. LC-MS/MS Analysis

First, 10 µg of eluates were subjected to in-solution tryptic digestion using a modified version of the single-pot solid-phase-enhanced sample preparation (SP3) protocol [100,101]. In total, three biological replicates were prepared, including recombinant wild-type *C. thermophilum*, *Ct*Naa15, and *Ct*Naa50 pull-out proteins (*n* = 3). Eluates were added to Sera-Mag Beads (Thermo Fisher Scientific, Waltham, MA, USA; #4515-2105-050250) in 10 µL of 15% formic acid and 30 µL of ethanol. The binding of proteins was achieved by shaking for 15 min at room temperature. SDS was removed by four subsequent washes with 200 µL of 70% ethanol. Proteins were digested overnight at room temperature with 0.4 µg of sequencing-grade modified trypsin (Promega, Madison, Wisconsin, USA; #V5111) in 40 µL of pH 8.4 HEPES/NaOH in the presence of 1.25 mM TCEP and 5 mM chloroacetamide (Sigma-Aldrich, Chemie GmbH, Taufkirchen, Germany; #C0267). The beads were separated and washed with 10 µL of an aqueous solution of 2% DMSO, and the combined eluates were dried down. The peptides were reconstituted in 10 µL of H_2_O and reacted for 1 h at room temperature with 80 µg of TMT10plex (Thermo Fisher Scientific, Waltham, MA, USA; #90111) [102] label reagent dissolved in 4 µL of acetonitrile. Excess TMT reagent was quenched by the addition of 4 µL of an aqueous 5% hydroxylamine solution (Sigma-Aldrich Chemie GmbH, Taufkirchen, Germany; #438227). The peptides were mixed and purified by a reverse-phase clean-up step (OASIS HLB 96-well µElution Plate, Waters Ltd., Wilmslow, UK; #186001828BA) following their analysis by LC-MS/MS on an Orbitrap Fusion Lumos mass spectrometer (Thermo Fisher Scientific, Waltham, MA, USA) as previously described [103]. To this end, peptides were separated using an Ultimate 3000 nano RSLC system (Dionex, Sunnyvale, CA, USA) equipped with a trapping cartridge (Precolumn C18 PepMap100, 5 mm, 300 μm i.d., 5 μm, 100 Å) and an analytical column (Acclaim PepMap 100, 75 × 50 cm C18, 3 mm, 100 Å) connected to a nanospray-Flex ion source. The peptides were loaded onto the trap column at 30 µL per min using solvent A (0.1% formic acid) and eluted using a gradient from 2 to 40% solvent B (0.1% formic acid in acetonitrile) over 2 h at 0.3 µL per min (all solvents were of LC-MS-grade). The Orbitrap Fusion Lumos was operated in positive ion mode with a spray voltage of 2.4 kV and capillary temperature of 275 °C. Full scan MS spectra with a mass range of 375–1500 m/z were acquired in profile mode using a resolution of 120,000, with a maximum fill time of 50 ms or a maximum of 4 × 10^5^ ions (AGC), and an RF lens setting of 30%. Fragmentation was triggered for 3 s of cycle time for peptide-like features with charge states of 2–7 on the MS scan (data-dependent acquisition). Precursors were isolated using the quadrupole with a window of 0.7 m/z and fragmented with a normalized collision energy of 38. Fragment mass spectra were acquired in profile mode and had a resolution of 30,000 in profile mode. The maximum fill time was set to 64 ms or an AGC target of 1 × 10^5^ ions. The dynamic exclusion was set to 45 s.

Acquired data were analyzed using IsobarQuant [104] and Mascot V2.4 (Matrix Science Ltd., London, UK) using a reverse UniProt FASTA *Chaetomium thermophilum* database including common contaminants, as previously described [71]. The following modifications were considered: carbamidomethyl (C, fixed), TMT10plex (K, fixed), acetyl (N-term, variable), oxidation (M, variable), and TMT10plex (N-term, variable). The mass error tolerance for full scan MS spectra was set to 10 ppm, and for MS/MS spectra it was set to 0.02 Da. A maximum of two missed cleavages were allowed. A minimum of two unique peptides with a peptide length of at least seven amino acids and a false discovery rate below 0.01 were required on the peptide and protein levels [105].

The raw output files of IsobarQuant (protein.txt files) were processed using the R programming language (ISBN 3-900051-07-0). Only proteins that were quantified with at least two unique peptides were considered for the analysis. A total of 748 proteins passed the quality control filters. Raw TMT reporter ion intensities (signal_sum columns) were first cleaned for batch effects using limma [106] and further normalized using vsn (variance stabilization normalization [107]). Proteins were tested for differential expression using the limma package. A protein was annotated as a hit with a false discovery rate (fdr) smaller than 0.05 and a fold-change of at least 100%.

Data were deposited to the ProteomeXchange Consortium via the PRIDE partner repository with the identifier PXD035320, and all identified proteins are listed in Supplementary Data S1.

### 4.14. C. thermophilum 80S Ribosome Preparation

*C. thermophilum* nontranslating 80S ribosome purification was performed with minor adaptations as previously described [108]. In brief, cells were grown at 90 rpm at 55 °C for 3 d, harvested by filtration, and frozen in liquid nitrogen. Cells were lysed in an MM400 cryogenic mixer mill (Retsch GmbH, Haan, Germany) at 30 s^−1^ for five minutes. The mycelium powder was resuspended in 20 mM pH 7.5 HEPES-KOH, 500 mM potassium acetate, 5 mM magnesium acetate, 2 mM DTT, 0.5 mM PMSF, and 1× protease inhibitor cocktail (PI; Serva Electrophoresis, Heidelberg, Germany). The lysate was cleared by centrifugation (55,000× *g*, JA25-50 rotor (Beckman Coulter GmbH, Krefeld, Germany), 35 min). Ribosomes were pelleted with a sucrose cushion (20 mM pH 7.5 HEPES-KOH, 500 mM potassium acetate, 1.5 M sucrose, 5 mM magnesium acetate, 2 mM DTT, and PI) at 125,000× *g* in a Ti-865 rotor (Thermo Fisher Scientific, Waltham, MA, USA) for 18 h and resuspended in 20 mM pH 7.5 HEPES-KOH, 50 mM potassium acetate, 5 mM magnesium acetate, 2 mM DTT, 0.5 mM PMSF, and PI. The ribosomes were then incubated with 1 mM neutralized puromycin and 1 mM GTP for 1 h at 30 °C. The ribosomes were further purified in a 15–40% sucrose gradient (20 mM pH 7.5 HEPES-KOH, 150 mM potassium acetate, 5 mM magnesium acetate, 15–40% sucrose, 2 mM DTT, 0.5 mM PMSF, and PI) at 60,000× *g* in a Surespin 630 rotor (Sorvall - Thermo Fisher Scientific, Waltham, MA, USA) for 15 h. Peak fractions containing *Ct*80S monosomes were pooled. Ribosomes were concentrated with an Amicon Ultra concentrator (100 kDa cutoff, Merck KGaA, Darmstadt, Germany) to 3.4 µM while exchanging the buffer to 20 mM pH 7.5 HEPES-KOH, 50 mM potassium acetate, 5 mM magnesium acetate, 2 mM DTT, and 0.5 mM PMSF. The quality was controlled by negative stain electron microscopy, and ribosomes were stored at −80 °C.

### 4.15. Flag-Tag Pull-Down Assay

Flag-tagged proteins were used as bait for *C. thermophilum* 80S ribosomes. All steps were performed with Flag buffer (20 mM pH 7.5 HEPES, 50 mM potassium acetate, 5 mM magnesium acetate, 50 mM NaCl), and ribosomes were buffer-exchanged using Zeba desalting columns (7 kDa cutoff, Thermo Fisher Scientific, Waltham, MA, USA). Then, 5 nmol *Ct*NatA (*Ct*Naa15-strep2/*Ct*Naa10-Flag), Flag-YFP-strep2, Flag-*Ct*Naa50_82-445_-strep2, Flag-*Ct*Naa50_82-289_-strep2, or buffer were loaded on 25 µL of anti-Flag M2 affinity gel (Sigma-Aldrich Chemie GmbH, Taufkirchen, Germany) for 45 min at 4 °C. Beads were washed twice with 500 µL of Flag buffer, 320 nM 80S ribosomes were incubated on the beads for 45 min at 4 °C, and unbound ribosomes were collected at 300× *g* for 2 min. Beads were washed three times with 500 µL of Flag buffer. Proteins and bound ribosomes were eluted with 75 µL of Flag buffer supplemented with 200 µg mL^−1^ Flag peptide (Sigma-Aldrich) at 300× *g* for 2 min. Samples from excess ribosomes and elutions were analyzed by SDS-PAGE using a 12% Nu-PAGE Bis-Tris gel (Invitrogen, - Thermo Fisher Scientific, Waltham, MA, USA), which was stained with Coomassie.

## Figures and Tables

**Table 1:** Catalytic parameters of Naa50 acetylation activity. Acetylation was performed at 30 °C, 25 °C (plant), or 37 °C (human) with MVNALE, MASS (plant), or MLGPE (human) peptides, respectively. GNAT indicates GNAT domain constructs, ΔN indicates the N-terminal deletion construct, and ΔC indicates the C-terminal deletion construct. * Values for human (*Hs*) and *Arabidopsis thaliana* (*At*) Naa50 were taken from [39,61]. Measurements were performed in triplicates and are presented as means ± SD.

Enzyme	K_m_ (µM)	k_cat_ (min^−1^)	k_cat_/K_m_ (min^−1^µM^−1^)	*Ct*Naa50_82-289_ Efficiency (%)
*Ct*Naa50_82-289_ (GNAT)	52.7 ± 8.6	36.4 ± 1.8	0.69 ± 0.12	100 ± 17
*Ct*Naa50_82-445_ (ΔN)	41.2 ± 6.1	23.9 ± 1.0	0.58 ± 0.09	84 ± 13
*Ct*Naa50_1-289_ (ΔC)	31.9 ± 4.4	16.3 ± 0.6	0.51 ± 0.07	74 ± 10
*Ct*Naa50_82-289_ Y190F	22.0 ± 4.0	5.6 ± 0.3	0.26 ± 0.05	38 ± 7
*Ct*Naa50_82-289_ H235A	15.3 ± 3.1	1.4 ± 0.1	0.09 ± 0.02	13 ± 3
*Nc*Naa50_93-287_ (GNAT)	146.9 ± 12.1	95.9 ± 3.0	0.65 ± 0.06	-
*At*Naa50*	201.9 ± 30.3	15.6 ± 1.2	0.08 ± 0.02	-
*Hs*Naa50*	4.62 ± 0.85	5.22 ± 0.23	1.13 ± 0.21	-

**Table 2:** Crystallographic data and refinement statistics. Data for the highest-resolution shell are in parenthesis.

	*Ct*Naa50_82-289_/CoA-Ac-MVNAL
**Data collection**	
Space group	P2_1_2_1_2_1_
Resolution (Å)	41.67–1.1 (1.14–1.1)
Unique reflection	92854 (9100)
a, b, c (Å)	36.94, 43.65, 140.07
α, β, γ (°)	90.0, 90.0, 90.0
R_merge_	0.066 (1.366)
R_pim_	0.019 (0.396)
Mean (I/σ(I))	18.01 (1.45)
Multiplicity	12.7 (12.5)
Completeness (%)	99.88 (99.66)
CC_1/2_	1 (0.631)
**Refinement**	
R_work_ (%)	14.42 (23.66)
R_free_ (%)	16.38 (25.37)
RMSD Bond length (Å)	0.008
RMSD Bond angle (°)	1.39
Ramachandran favored (%)	98.14
Ramachandran allowed (%)	1.86
Ramachandran outliers (%)	0.0
Rotamer outliers (%)	0.53
Clashscore	1.60
Average B factor (Å^2^)	20.45
Protein	18.29
Ligands	30.29
Solvent	33.11
**PDB identifier**	7OJU

## Data Availability

Coordinates and structure factors for *Ct*Naa50_82-289_/CoA-Ac-MVNAL have been deposited at the Protein Data Bank under the accession code 7OJU. The mass spectrometry proteomics data have been deposited to the ProteomeXchange Consortium via the PRIDE [109] partner repository with the dataset identifier PXD035320. Further data supporting the findings of this study are available from the corresponding author upon reasonable request.

## Supplementary Materials

The following supporting information can be downloaded at: https://www.mdpi.com/article/10.3390/ijms231810805/s1.
