# Supplementary material for: Extended N-Terminal Acetyltransferase Naa50 in Filamentous Fungi Adds to Naa50 Diversity
Source: Int J Mol Sci. 2022 Sep 16;23(18):10805. doi: 10.3390/ijms231810805 (PMC9500918; doi:10.3390/ijms231810805)
Supplement: Supplementary file 1 [file ijms-23-10805-s001.zip › ijms-1877136-supplementary/Supplementary_materials.pdf]

Supplementary materials

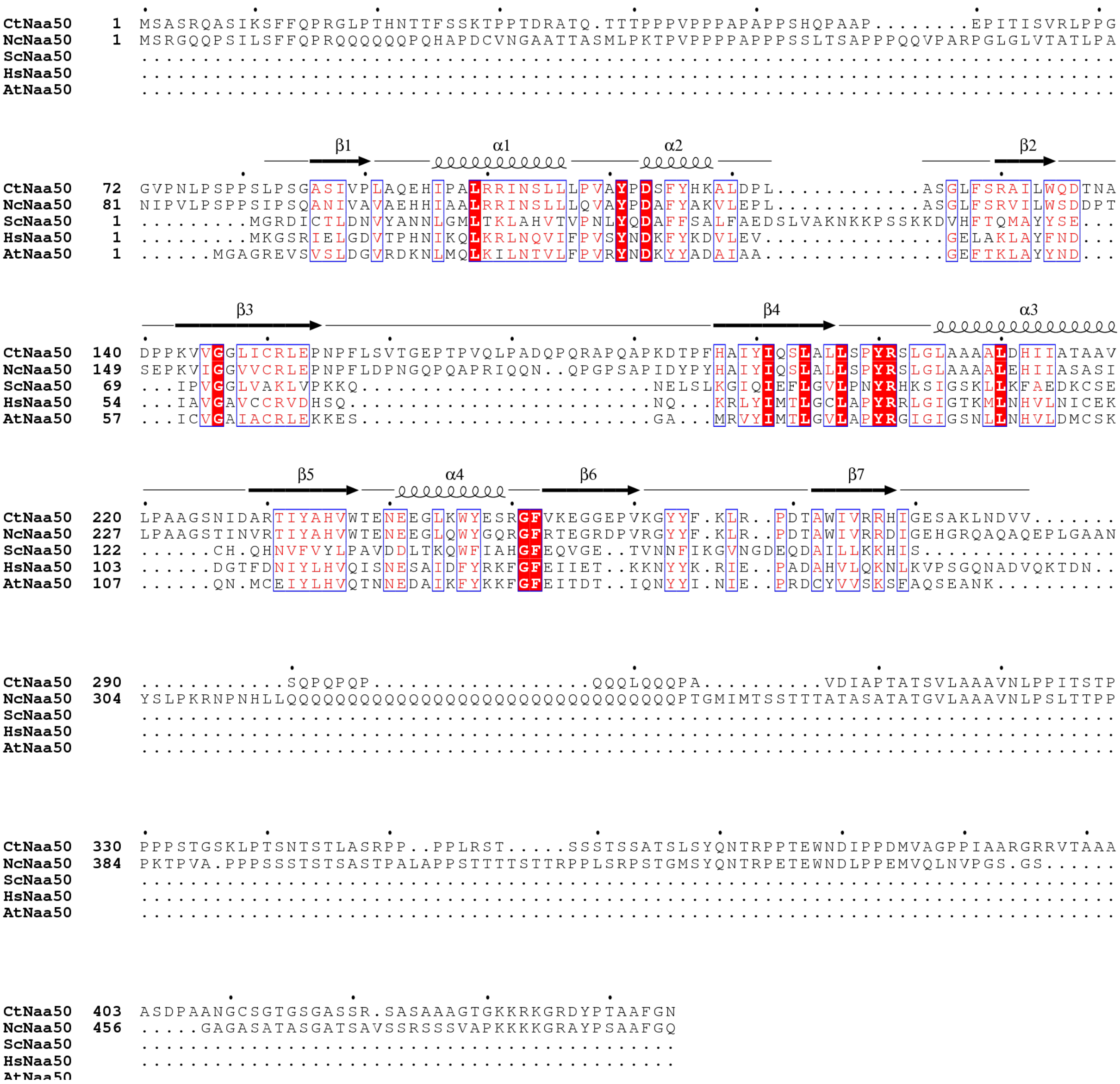

Supplementary Figure S1. Multiple sequence alignment of Naa50 from different species. Naa50 from *Chaetomium thermophilum* (*Ct*), *Neurospora crassa* (*Nc*), *Saccharomyces cerevisiae* (*Sc*), *Homo sapiens* (*Hs*), *and Arabidopsis thaliana* (*At*) show an overall high conservation for the GNAT-domain (framed by arrows). Secondary structure elements and boundaries from the GNAT-domain construct *Ct*Naa50$_{82\text{-}289}$ are shown on top. *C. thermophilum* and *N. crassa* Naa50 have elongated termini. Fully conserved residues are represented as white letters in red boxes. Similarities are shown with red letters in blue frames. The sequence alignment was performed using Clustal Omega and visualized using ESPript 3.0 [56; 57].

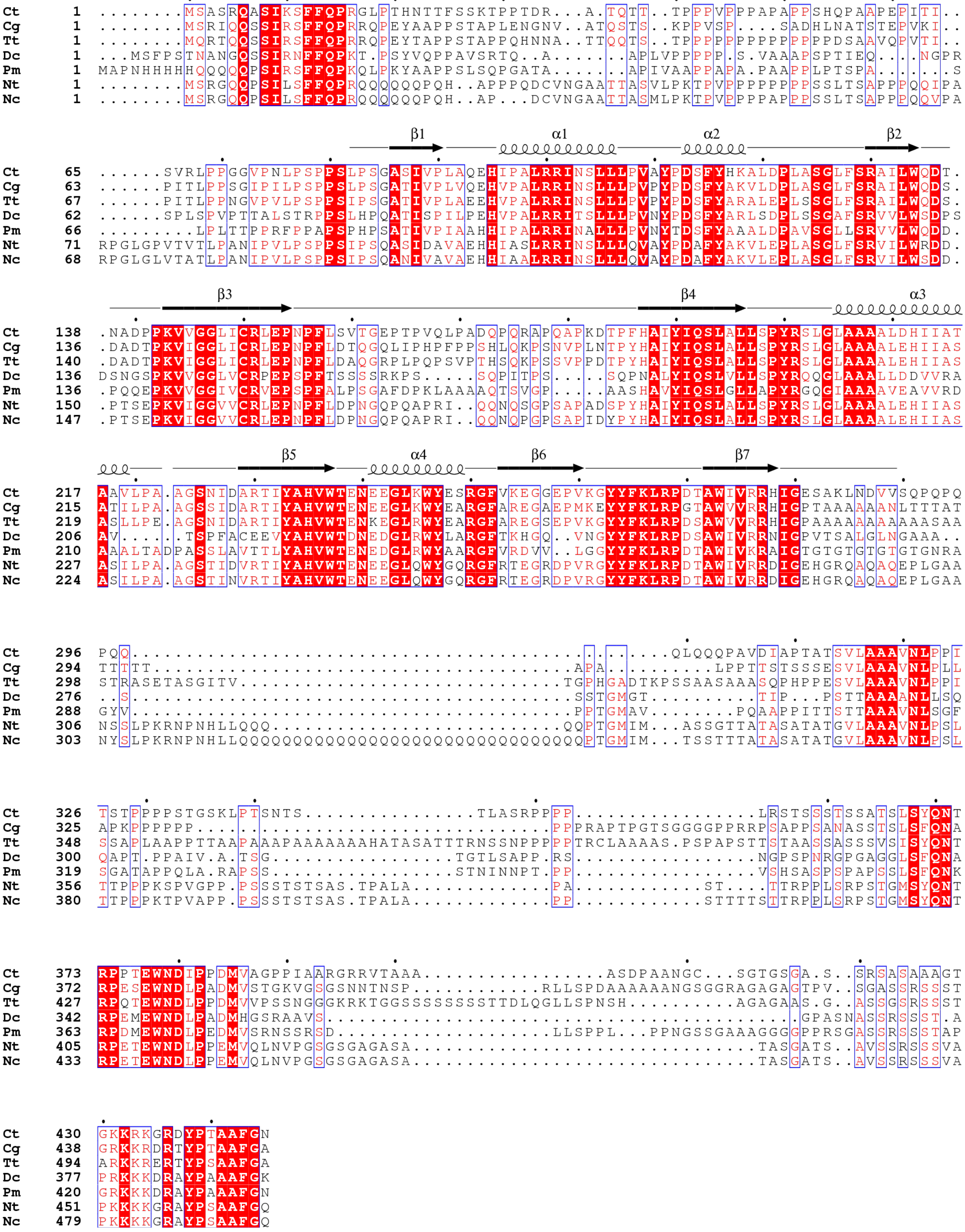

Supplementary Figure S2. Multiple sequence alignment of elongated Naa50. Naa50 candidates from *Chaetomium thermophilum* (*Ct*), *Chaetomium globosum* (*Cg*), *Thermothelomyces thermophilus* (*Tt*), *Daldinia childiae* (*Dc*), *Phaeoacremonium minimum* (*Pm*), *Neurospora tetrasperma* (*Nt*), and *Neurospora crassa* (*Nc*) show an overall high conservation. Secondary structure elements and boundaries from the GNAT-domain construct $CtNaa50_{82\text{-}289}$ are shown on top. Fully conserved residues are represented as white letters in red boxes. Similarities are shown with red letters in blue frames. The sequence alignment was performed using Clustal Omega and visualized using ESPript 3.0 [56; 57].

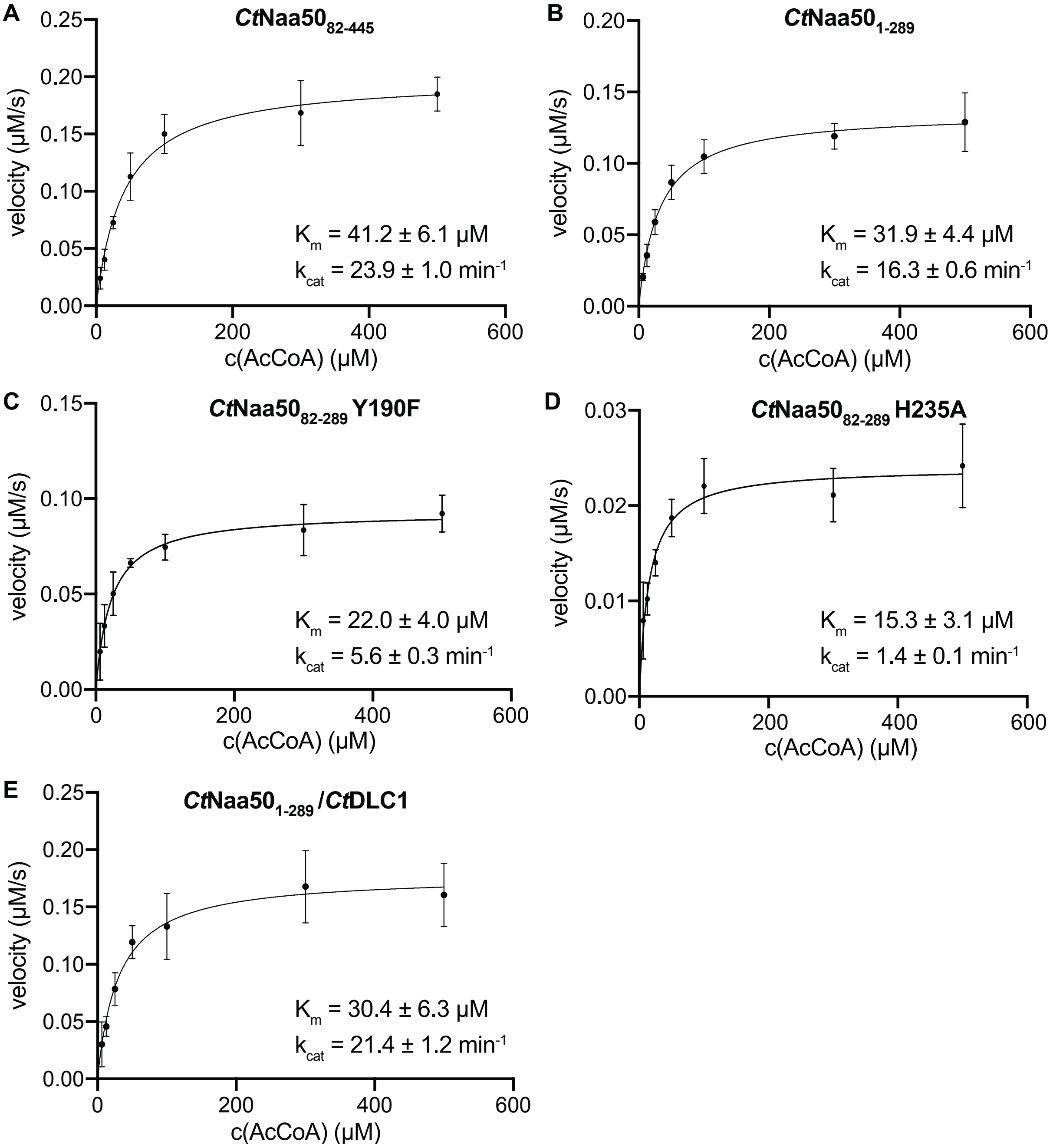

Supplementary Figure S3. Michaelis-Menten kinetics for the acetylation of MVNALE by several *Ct*Naa50 variants with increasing AcCoA concentrations. (**A**) *Ct*Naa50$_{82\text{-}445}$ (ΔN) and (**B**) *Ct*Naa50$_{1\text{-}289}$ (ΔC) have similar parameters and feature only a slightly reduced activity compared to the GNAT construct *Ct*Naa50$_{82\text{-}289}$. (**C**) *Ct*Naa50$_{82\text{-}289}$ Y190F and (**D**) *Ct*Naa50$_{82\text{-}289}$ H235A mutants have a reduced activity. (**E**) *Ct*Naa50$_{1\text{-}289}$/*Ct*DLC1 complex acetylates MVNALE similar to *Ct*Naa50$_{1\text{-}289}$ alone. All measurements were performed in triplicates and are presented as mean ± SD.

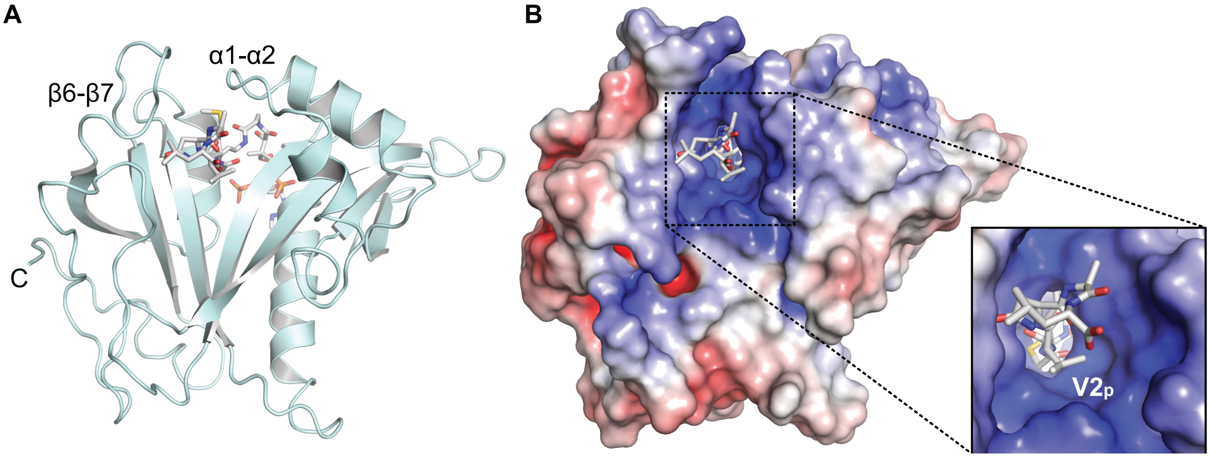

Supplementary Figure S4. *Ct*Naa50$_{82\text{-}289}$ binding pocket for V2$_P$ has a positive surface potential. (**A**) The structure of *Ct*Naa50$_{82\text{-}289}$ (teal) in complex with CoA-Ac-MVNAL in cartoon and sticks representation with view on the substrate peptide binding site. (**B**) Electrostatic surface potential representation of *Ct*Naa50$_{82\text{-}289}$ with focus on the substrate peptide binding site and zoom-in on the substrate peptide's second position V2$_P$ with surroundings. Electrostatics are represented in a gradient from negative (red, -5 kT/e) to positive (blue, +5 kT/e).

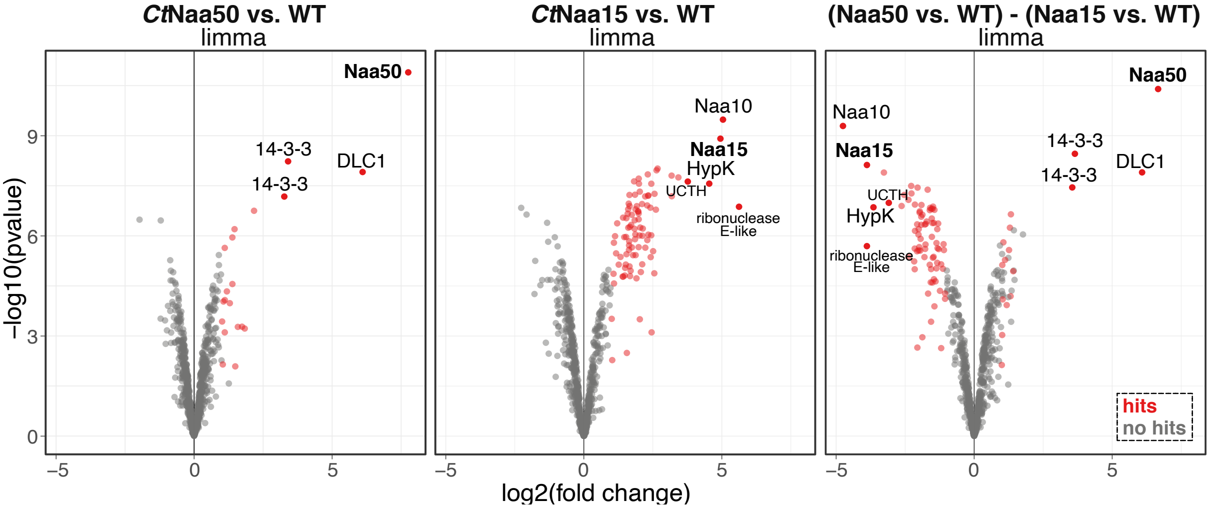

Supplementary Figure S5. Volcano plots of *Ct*Naa50 and *Ct*Naa15 pull-outs versus wild type control from TMT-labeled LC-MS/MS. *Ct*Naa50 (left) pull-outs result in additional enrichment of putative dynein light chain 1 protein (NCBI identifier CTHT_0058240), and two hypothetical 14-3-3 proteins (CTHT_0054160, CTHT_0054410). *Ct*Naa15 (middle) pull-outs result in enrichment of Naa10 (CTHT_0063490), HypK (CTHT_0058830), ubiquitin carboxyl-terminal hydrolase-like protein (UCTH, CTHT_0063590), and an uncharacterized protein (CTHT_0074400; ribonuclease E-like). The comparison between the two pull-outs (right) highlights that NatA subunits and Naa50 are exclusively found in their respective pull-outs, indicating absent interaction between *Ct*Naa50 and the *Ct*NatA subunits. Bold protein labels indicate the tagged proteins. Wild type (WT) data represent mock-purified proteins without any tag to account for the background level of unspecifically bound proteins. All measurements were performed in triplicates.

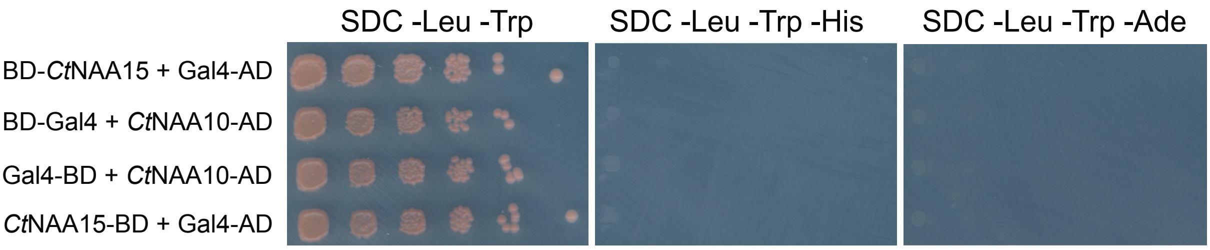

Supplementary Figure S6. Yeast two-hybrid *Ct*NAA10 and *Ct*NAA15 self-activation controls. There is no self-activation observable for *Ct*NAA15 or *Ct*NAA10 with the respective Gal4 activation (AD) or binding domains (BD) as negative controls. Growth on SDC -Leu/ -Trp indicates successful transformation.

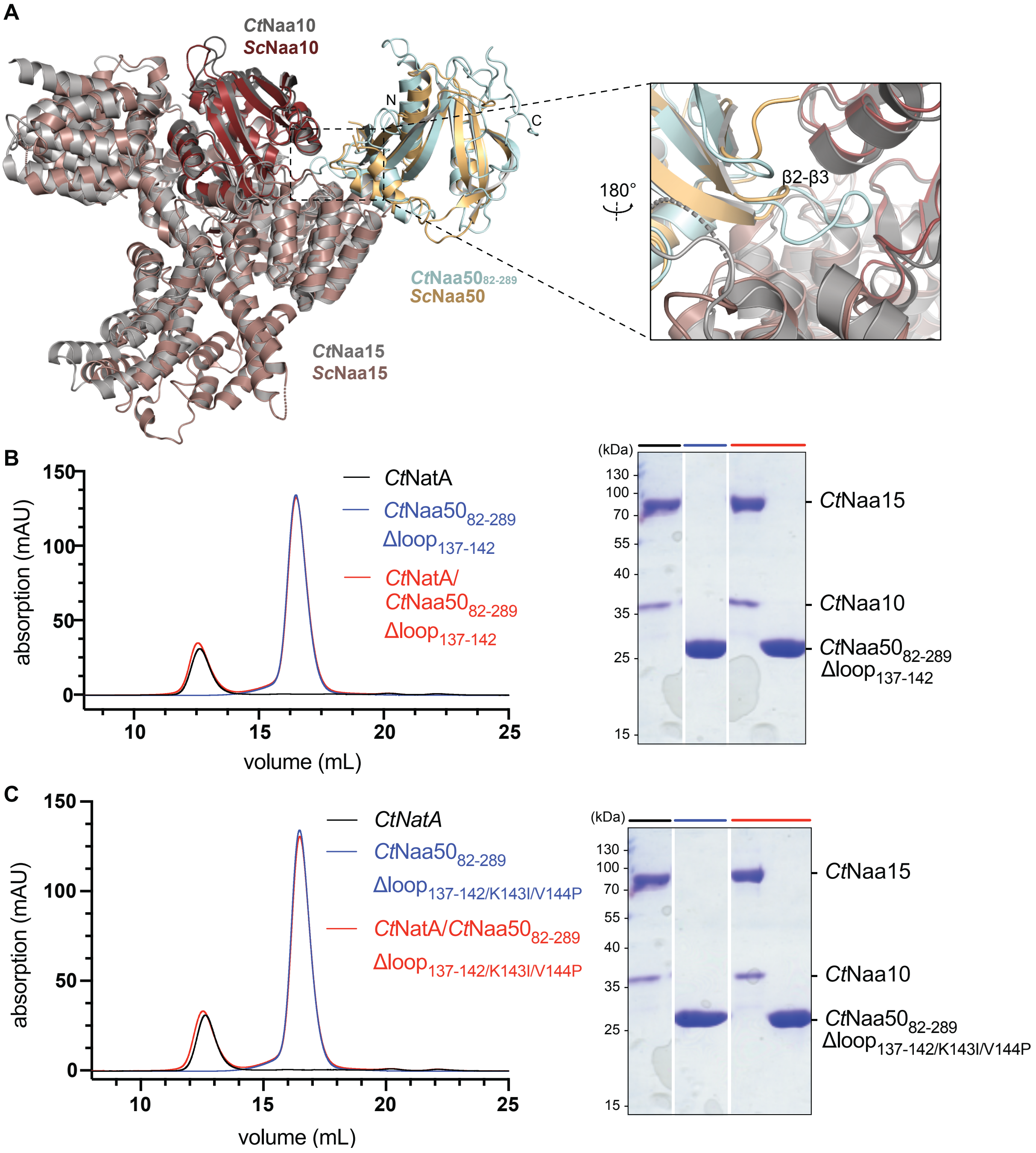

Supplementary Figure S7. *Ct*Naa50$_{82\text{-}289}$ does not bind to *Ct*NatA. (**A**) Superimposition of *Ct*Naa50$_{82\text{-}289}$ on *Sc*Naa50, as well as *Ct*NatA (PDB identifier 5NNP, [29]) on *Sc*NatA in the *Sc*NatE complex (PDB identifier 4XNH) shows that – given a similar interaction as in *Sc*NatE - the longer *Ct*Naa50$_{82\text{-}289}$ loop β2-β3 would clash with NatA. (**B**) *Ct*Naa50$_{82\text{-}289}$ Δloop$_{137\text{-}142}$ mutant does not co-elute with *Ct*NatA from an analytical size-exclusion column (Superdex 200 10/300 GL, GE Healthcare). (**C**) *Ct*Naa50$_{82\text{-}289}$ Δloop$_{137\text{-}142/K143I/V144P}$ mutant does not co-elute with *Ct*NatA from an analytical size-exclusion column (Superdex 200 10/300 GL, GE Healthcare). Mutating *Ct*Naa50$_{82\text{-}289}$ loop β2-β3 does not lead to NatA binding.

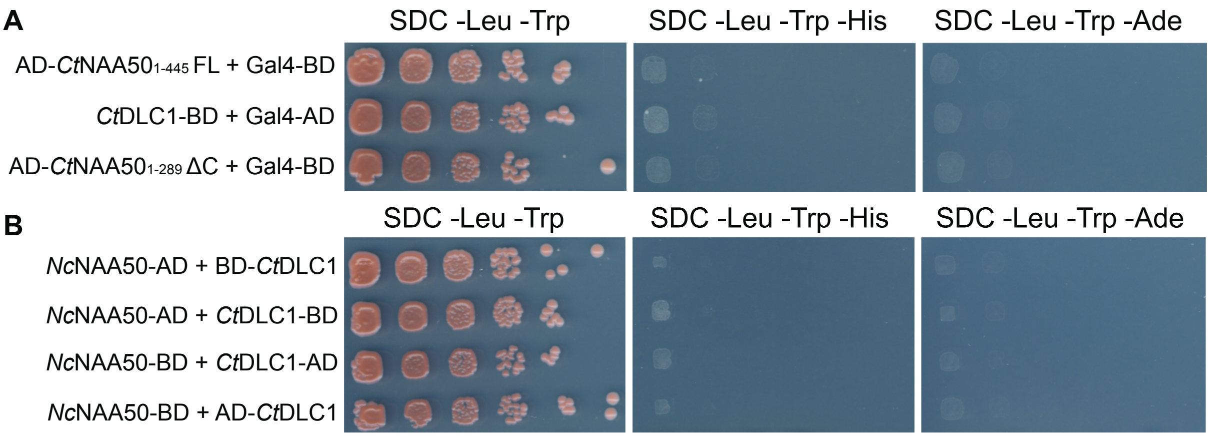

Supplementary Figure S8. Yeast two-hybrid *Ct*NAA50 and *Ct*DLC1 self-activating controls and *Nc*NAA50/*Ct*DLC1 interaction assays. (**A**) There is no self-activation observable for *Ct*NAA50$_{1-445}$ (FL), *Ct*NAA50$_{1-289}$ (ΔC), or *Ct*DLC1 with the respective Gal4 activation (AD) or binding domains (BD) as negative controls. (**B**) *Nc*NAA50$_{1-494}$ (full length) does not interact with *Ct*DLC1 in various orientations of Gal4 activation (AD) or binding domains (BD). Growth on SDC -Leu/ -Trp indicates successful transformation.

Supplementary Table S1. List of used oligonucleotides for cloning, and mutagenesis. Oligonucleotide sequences are in 5′-3′ orientation. Names of the oligonucleotides also list their features. F = forward primer, R = reverse primer. Restriction enzymes (e.g., NcoI) and affinity tags for purification (His = hexahistidine, strep = strep2, Flag tags) were introduced. Δ identifies amino acid deletion constructs, either N-terminal, C-terminal or internal deletions. noStop oligonucleotides were used to remove the stop codon. polyA mutagenesis primers were used to mutate amino acid patches completely to alanine residues.

**Name with features**	**Sequence 5′-3′**
**Cloning**	
*Ct*DLC1_His_BamHI_R	CGCGGATCCCTAATGGTGATGGTGATGGTGCTGGGTCTTGAACAGCAAGATG
*Ct*DLC1_BamHI_R	CGCGGATCCCTACTGGGTCTTGAACAGCAAG
*Ct*DLC1_NcoI_F	CATGCCATGGGTAAGAGCGACGAGAAGAAGAGC
*Ct*DLC1_noStop_BamHI_R	CGCGGATCCCTGGGTCTTGAACAGCAAGATG
*Ct*NAA10_BamHI_R	CGCGGATCCCTATGTATGCTTACTCTCATCCCTC
*Ct*NAA10_Flag_BamHI_R	GCGGGATCCTTACTTGTCGTCATCGTCTTTGTAGTCTGTATGCTTACTCTCATCCCTC
*Ct*NAA10_NcoI_F	CATGCCATGGACATCCGCCTCCTCC
*Ct*NAA15_NcoI_F	CATGCCATGGGCCCGCAGCCACTGAGTACG
*Ct*NAA15_noStop_BamHI_R	CGCGGATCCGGCGCCAGGCACACCAAC
*Ct*NAA15_strep_BamHI_R	GCGGGATCCTTACTTTTCGAACTGCGGGTGGCTCCAGGCGCCAGGCACACCAAC
*Ct*NAA15_BamHI_R	CGGGATCCGGCGCCAGGCACACCAAC
*Ct*NAA50_BamHI_R	CGCGGATCCTTAATTCCCAAACGCCGCAGTAG
*Ct*NAA50_ΔC156_BamHI_R	CGCGGATCCTTACACAACATCATTCAGCTTCGCG
*Ct*NAA50_ΔC156_His_BamHI_R	CGCGGATCCTTAATGGTGATGGTGATGGTGCACAACATCATTCAGCTTCGCG
*Ct*NAA50_ΔN81_NcoI_F	CATGCCATGGGACTCCCCTCTGGCGCAAGC
*Ct*NAA50_His_BamHI_R	CGCGGATCCTTAATGGTGATGGTGATGGTGATTCCCAAACGCCGCAGTAG
*Ct*NAA50_NcoI_F	CATGCCATGGGATCTGCCTCCCGCCAGGC
*Ct*NAA50_ΔC156_strep_BamHI_R	CGCGGATCCTTACTTTTCGAACTGCGGGTGGCTCCACACAACATCATTCAGCTTCGCG
*Ct*NAA50_ΔN81_Flag_NcoI_F	CATGCCATGGACTACAAAGACGATGACGACAAGCTCCCCTCTGGCGCAAGC
*Ct*NAA50_FL_Flag_NcoI_F	CATGCCATGGACTACAAAGACGATGACGACAAGATGTCTGCCTCCCGCCAGG
*Ct*NAA50_FL_strep_BamHI_R	CGCGGATCCTTACTTTTCGAACTGCGGGTGGCTCCAATTCCCAAACGCCGCAGTAGG
*Ct*NAA50_NdeI_F	GGAATTCCATATGTCTGCCTCCCGCCAGGC
*Nc*NAA50_BamHI_R	CGGGATCCCTACTGCCCAAAAGCAGCACTAGG
*Nc*NAA50_NcoI_F	CATGCCATGGCTTCGCGTGGTCAACAACCCTC
*Nc*NAA50_ΔC206_His_BamHI_R	CGGGATCCCTAATGGTGATGGTGATGGTGTCCAATGTCTCTACGCACAATCC
*Nc*NAA50_ΔN92_NcoI_F	CATGCCATGGCTTCGCAAGCCAACATAGTCGC
YFP_Flag_NcoI_F	CATGCCATGGACTACAAAGACGATGACGACAAGATGGGCAAAGTGAGCAAGGG
YFP_strep_BamHI_R	GCGGGATCCTTACTTTTCGAACTGCGGGTGGCTCCACTTGTACAGCTCGTCCATGC
**Mutagenesis**	
*Ct*NAA50_Δ137-142+_F	AATTCTCTGGCAAGACATACCGGTCGGCGGTCTGATCT
*Ct*NAA50_Δ137-142+_R	AGATCAGACCGCCGACCGGTATGTCTTGCCAGAGAATT
*Ct*NAA50_Δ137-142_F	GACCGCCGACCACCTTGTCTTGCCAGAGAATT
*Ct*NAA50_Δ137-142_R	AATTCTCTGGCAAGACAAGGTGGTCGGCGGTC
*Ct*NAA50_DLC1motif_polyA_F	AAAACCCCTCCCACAGACGCCGCAGCTGCAGCCGCCACACCGCCGCCTGTCC
*Ct*NAA50_DLC1motif_polyA_R	GGACAGGCGGCGGTGTGGCGGCTGCAGCTGCGGCGTCTGTGGGAGGGGTTTT
*Ct*NAA50_H235A_F	TGCAAGGACGATTTATGCCGCTGTGTGGACAGAAAATGAG
*Ct*NAA50_H235A_R	CTCATTTTCTGTCCACACAGCGGCATAAATCGTCCTTGCA
*Ct*NAA50_Y190F_F	CCCCGTTTCATGCGATTTTTATCCAGAGTTTGGCT
*Ct*NAA50_Y190F_R	AGCCAAACTCTGGATAAAAATCGCATGAAACGGGG

Supplementary Table S2. List of plasmids. Plasmid backbones with their features are listed with different inserts for the coding sequences of all constructs. Superscript R identifies resistances. Δ represents deletion constructs. His_6 is a hexahistidine tag. Plasmids follow the convention plasmid::insert in the main text (e.g., pET21d::*Ct*DLC1).

Plasmid	Features	Inserts
pET21d	*E. coli* expression under T7 promoter, Amp^R	*Ct*DLC1, *Ct*DLC1-His_6, *Ct*Naa10-Flag
pET24d	*E. coli* expression under T7 promoter, Kan^R	*Ct*Naa10-His_6, *Ct*Naa15, *Ct*$Naa50_{82\text{-}289}$-H235A-His_6, *Ct*$Naa50_{82\text{-}289}$-His_6, *Ct*$Naa50_{82\text{-}289}$-Y190F-His_6, *Ct*$Naa50_{82\text{-}289}$-Δ137-142_K143I_V144P-His_6, *Ct*$Naa50_{82\text{-}289}$-Δ137-142-His_6, *Ct*$Naa50_{82\text{-}445}$-His_6, *Nc*$Naa50_{93\text{-}287}$-His_6
pETNHis1a	*E. coli* expression under T7 promoter, N-terminal hexahistidine tag, TEV protease cleavage site, Kan^R	*Ct*Naa15-strep2, Flag-*Ct*$Naa50_{82\text{-}289}$-strep2, Flag-*Ct*$Naa50_{82\text{-}445}$-strep2, Flag-YFP-strep2
pG4BDN22	*S. cerevisiae* Y2H, ADH1 promoter, NLS signal, N-terminal GAL4 binding domain (BD), Amp^R and TRP1 nutritional marker (Trp^+)	*Ct*DLC1, *Ct*Naa15, *Ct*Naa50, *Ct*Naa50-polyA:34-39, *Ct*$Naa50_{1\text{-}289}$, *Ct*$Naa50_{82\text{-}289}$, *Ct*$Naa50_{82\text{-}445}$
pG4BDC22	*S. cerevisiae* Y2H, ADH1 promoter, NLS signal, C-terminal GAL4 binding domain (BD), Amp^R and TRP1 nutritional marker (Trp^+)	*Ct*DLC1, *Ct*Naa10, *Ct*Naa15, *Ct*Naa50, *Ct*Naa50-polyA:34-39, *Ct*$Naa50_{1\text{-}289}$, *Ct*$Naa50_{82\text{-}289}$, *Ct*$Naa50_{82\text{-}445}$, *Nc*Naa50
pG4ADHAN111	*S. cerevisiae* Y2H, ADH1 promoter, NLS signal, N-terminal GAL4 activation domain (AD), Amp^R and LEU2 nutritional marker (Leu^+)	*Ct*DLC1, *Ct*Naa15, *Ct*Naa50, *Ct*Naa50-polyA:34-39, *Ct*$Naa50_{1\text{-}289}$, *Ct*$Naa50_{82\text{-}289}$, *Ct*$Naa50_{82\text{-}445}$
pG4ADC111	*S. cerevisiae* Y2H, ADH1 promoter, NLS signal, C-terminal GAL4 activation domain (AD), Amp^R and LEU2 nutritional marker (Leu^+)	*Ct*DLC1, *Ct*Naa10, *Ct*Naa50, *Ct*Naa50-polyA:34-39, *Ct*$Naa50_{1\text{-}289}$, *Ct*$Naa50_{82\text{-}289}$, *Ct*$Naa50_{82\text{-}445}$, *Nc*Naa50
pRS426	*S. cerevisiae* Y3H, ADH1 promoter, NLS signal, Amp^R and URA3 nutritional marker (Ura^+)	*Ct*Naa10
pMT929NHis1a	*S. cerevisiae* expression under GAL1/GAL10 promoter, N-terminal hexahistidine tag, TEV protease cleavage site, Amp^R and TRP1, LEU2 nutritional markers (Trp^+, Leu^+)	*Ct*$Naa50_{1\text{-}289}$
pRSF-duet-FTpA	*C. thermophilum* expression under actin promoter, C-terminal Flag-tag > TEV protease site > protein A-tag, Amp^R and $terbinafine^R$ (erg1 gene)	*Ct*Naa15
pRSF-duet-pAFT	*C. thermophilum* expression under actin promoter, N-terminal protein A-tag > TEV protease site > Flag-tag, Amp^R and $terbinafine^R$ (erg1 gene)	*Ct*Naa50

Supplementary Table S3. List of *Chaetomium thermophilum* strains. Strain names, genotypes, and their source are listed below.

Strain	Genotype	Source
C. thermophilum (La Touche)	wild type: DSMZ#1495	[88]
C. thermophilum FTpA-NAA15	*P-ACTIN:ERG1:T-GPD; P-NAA15-FTpA:T-GPD*	this study
C. thermophilum NAA50-FTpA	*P-ACTIN:ERG1:T-GPD; P-FTpA-NAA50:T-GPD*	this study

References in supplementary materials follow the numbering in the main article:

29. Weyer, F.A., Gumiero, A., Lapouge, K., Bange, G., Kopp, J., and Sinning, I. (2017). Structural basis of HypK regulating N-terminal acetylation by the NatA complex. Nat Commun *8*, 15726. 10.1038/ncomms15726

56. Madeira, F., Park, Y.M., Lee, J., Buso, N., Gur, T., Madhusoodanan, N., Basutkar, P., Tivey, A.R.N., Potter, S.C., Finn, R.D., *et al.* (2019). The EMBL-EBI search and sequence analysis tools APIs in 2019. Nucleic Acids Res *47*, W636-W641. 10.1093/nar/gkz268

57. Robert, X., and Gouet, P. (2014). Deciphering key features in protein structures with the new ENDscript server. Nucleic Acids Res *42*, W320-324. 10.1093/nar/gku316

88. Amlacher, S., Sarges, P., Flemming, D., van Noort, V., Kunze, R., Devos, D.P., Arumugam, M., Bork, P., and Hurt, E. (2011). Insight into structure and assembly of the nuclear pore complex by utilizing the genome of a eukaryotic thermophile. Cell *146*, 277-289. 10.1016/j.cell.2011.06.039
